# Identification and Characterization of a Neutral Locus for Knock-in Purposes in *C. parapsilosis*

**DOI:** 10.3389/fmicb.2020.01194

**Published:** 2020-06-05

**Authors:** Tibor Nemeth, Csaba Papp, Csaba Vagvolgyi, Tanmoy Chakraborty, Attila Gacser

**Affiliations:** ^1^Department of Microbiology, University of Szeged, Szeged, Hungary; ^2^MTA-SZTE Lendület Mycobiome Research Group, University of Szeged, Szeged, Hungary

**Keywords:** *Candida parapsilosis*, knock-in, overexpression, reintegration, genetic manipulation, GFP

## Abstract

Invasive fungal infections caused by *Candida* species affect approximately 700,000 people worldwide resulting in 300,000 deaths annually. Besides *Candida albicans*, other members of the genus have gained relevance in the last two decades, including *C. parapsilosis* whose incidence is particularly high amongst low birth weight neonates. To investigate the virulence properties of this pathogen several techniques have been developed for generating knock-out mutants, however, no target locus for knock-in approaches have been published so far. Here we report CpNEUT5L (N5L), an intergenic locus in *C. parapsilosis*, and introduce an integrative Gateway^TM^ and a classical ligation based replacement plasmid to target it with. As a proof of principle, we fluorescently tagged laboratory and prototroph strains and established that this locus is also suitable for reintegration purposes. We concluded that GFP-expressing constructs integrated into this region provide strong, homogenous fluorescent signals while alteration of this locus affects neither the growth of the mutants in liquid or on solid media, even in the presence of different stressors, nor their basic virulence properties. Hence, our findings demonstrate that N5L is a highly effective neutral locus for knock-in approaches in *C. parapsilosis*.

## Introduction

Fungal infections represent a global threat with nearly one billion cases per year ranging from mild superficial to invasive infections with high mortality rates (Pfaller and Diekema, 2007; Bongomin et al., 2017). These latter diseases are most commonly associated with *Candida* species, especially amongst patients with defective immune system due to cancer treatment, HIV infection, or neutropenia, but prolonged hospitalizations and the use of catheters and other medical devices are also established as predisposing factors (Puzniak et al., 2004; Cheng et al., 2005; Li et al., 2017). Amongst these opportunistic pathogens, *Candida albicans* has long been known as the dominant etiologic agent within the genus associated with disease; however, according to recent literature, a remarkable shift in the epidemiology toward the so-called non-*albicans Candida* species (*C. glabrata*, *C. parapsilosis*, *C. tropicalis*, *C. krusei* and more recently *C. auris*) has occurred (Yapar, 2014; Cleveland et al., 2015; Cortegiani et al., 2018; Kothalawala et al., 2019). Some authors have identified *C. parapsilosis* as the second most frequently associated *Candida* species with bloodstream infections, and it has been reported as the major cause of invasive candidiasis in low and very low birth weight neonates (Pappas, 2006; Pammi et al., 2013; Jayaweera and Reyes, 2018). *C. parapsilosis* and *C. albicans* share some attributes as they are both diploid and both belong to the CUG clade, meaning that during translation the triplet CUG results in the incorporation of serine rather than leucine. Notably, they differ significantly in many aspects, including their resistance to antibiotics, recognition by host immune cells and filamentation ability, and *C. albicans* requires colonization before infection while *C. parapsilosis* does not (Nucci and Anaissie, 2001; Miranda et al., 2009; Tóth et al., 2013; Lin et al., 2015; Tóth et al., 2017). All these observations strengthen the hypothesis that these two pathogens are different by nature, and clinical and basic science findings regarding *C. albicans* are not necessarily valid for *C. parapsilosis*.

To better understand the physiology and pathogenicity of *C. parapsilosis*, several molecular tools have been applied that primarily focus on eliminating gene products, such as the *caSAT1* flipper, or the double auxotrophic complementation technique, and more recently the CRISPR/Cas9 system (Ding and Butler, 2007; Gácser et al., 2007; Holland et al., 2014; Lombardi et al., 2017). However, while these methods have revealed important characteristics of the physiology and virulence attributes of *C. parapsilosis*, our potential to deeply study the fungus remains limited due to the lack a complete set of molecular tools, such as those already available for *C. albicans*. For instance, for our virulence studies, it was essential to create an effective pipeline through which we could facilitate the reintegration workflow of our knock-out mutant collection and generate GFP-tagged derivatives of laboratory and also clinical strains to follow their interactions with host immune cells (Tóth et al., 2018). This effort, however, was complicated due to the absence of a characterized locus for knock-in approaches and a lack of compatible vectors. Recently a protein tagging method was described by Gonia and coworkers, where one allele of the ortholog of *C. albicans* enolase 1 was fluorescently labeled in *C. parapsilosis* (Gonia et al., 2017). Nonetheless, the goal of this work was to provide a tool for protein tagging in this species and was presented as a proof of principle rather than introducing a general locus for accepting exogenous sequences. Accordingly, we aimed to identify a genomic region suitable for acquiring such expression constructs, and provide a set of plasmids that are feasible for these purposes in *C. parapsilosis*. We targeted *CpRP10*, the ortholog or *RPS1* of *C. albicans*, and CpNEUT5L (N5L), the ortholog of the recently reported intergenic neutral locus of *C. albicans* (NEUT5L), with GFP expression constructs and, surprisingly, only the latter resulted in homogenous GFP expression in the population (Gerami-Nejad et al., 2013; Legrand et al., 2018). To target N5L, we developed two different set of plasmids. One is a derivative of the Gateway^TM^ compatible integrative vector (pCA-DEST1300) the other encodes a classical replacement cassette (pNRVL) (Legrand et al., 2018). Both the Gateway^TM^ and the pNRVL were equipped with either an auxotrophic marker *LEU2* from *C. maltosa*, or genes providing resistance to the dominant selectable marker nourseothricin (NTC) to make these plasmids applicable not only in laboratory but also in prototroph strains. Additionally, we generated an *ade2^–/–^* knock-out mutant and successfully applied an *ADE2* reintegration construct targeting N5L. Integration of expression constructs into this locus is a suitable approach for fluorescently tagged strains and it also facilitates the reintegration of NTC sensitive knock-out mutants. Integration of foreign DNA did not have any effect on the viability or on the basic virulence attributes of the transformants, making N5L an ideal locus to target with heterologous sequences in *C. parapsilosis*.

## Materials and Methods

Strains used in this study are listed in Supplementary Table S1.

Oligonucleotides used in this study are listed in Supplementary Table S2.

Plasmid editing (plasmids used in this study are listed in Supplementary Table S3).

To create a *C. parapsilosis* compatible destination plasmid pCA-DEST1300 was used (Legrand et al., 2018). The upstream and downstream regions of *CpRP10* (CPAR2_110290) ORF were amplified separately from genomic DNA of *C. parapsilosis* CLIB214 with primer pairs CpRP10UpMluIF – CpRP10UpStuIR and CpRP10DownStuIF – CpRP10DownSpeIR, respectively. Fragments were isolated and joined by fusion PCR by using CpRP10UpMluIF and CpRP10DownSpeIR. The pCA-DEST1300 was digested with *Mlu*I/*Spe*I and the isolated backbone was ligated with the *Mlu*I/*Spe*I digested *CpRP10* amplicon. The URA3 selection marker of the resulting plasmid was replaced with *C. maltosa LEU2* by amplifying the marker from pSN40 with primerpairs CmLEU2SacIF and CmLEU2SpeIR (Noble and Johnson, 2005; Holland et al., 2014). The plasmid backbone and amplicon were digested with *Spe*I and *Sac*I, isolated, and ligated to create pDCpOE-L-RP10^∗^. The *Eco*RV site lying downstream from attR2 was replaced to *Nhe*I by amplifying a region of pDCpOE-L-RP10^∗^ with primers pDESTClaIF and pDESTecorv-NsiIR. The amplicon and pDCpOE-L-RP10^∗^ were digested with *Cla*I/*Nsi*I and ligated to gain pDCpOE-L-RP10.

To change the *CpRP10* target locus, CpNEUT5L upstream and downstream sequences were amplified from *C. parapsilosis* CLIB214 genomic DNA with primer pairs CpNEUT5LUpMluIF – CpNEUT5LUpStuIR and CpNEUT5LDownStuIF – CpNEUT5LDownSpeIR. The two fragments were isolated then fused together by PCR using primers CpNEUT5LUpMluIF and CpNEUT5LDownSpeIR. The *CpRP10* sequence was released from pDCpOE-L-RP10 by *Mlu*I/*Spe*I digestion and the plasmid backbone was ligated with *Mlu*I/*Spe*I digested CpNEUT5L fusion product to generate pDCpOE-L-N5L.

Replacement of the *LEU2* selection marker was performed by amplifying the *caNAT1* marker from plasmid pMG2120 with the primer pair NAT1SpeIF – NAT1SacIR (Shen et al., 2005; Gerami-Nejad et al., 2012). The amplicon and pDCpOE-L-N5L were subsequently digested with *Sac*I and *Spe*I, then ligated to create pDCpOE-N-N5L.

CpNEUT5L Upup and Downdown regions were amplified with primer pairs CpNEUT5LUpupApaIStuIF – CpNEUT5LUpupfusionR and CpNEUT5LDodofusionF – CpNEUT5LDodoStuIEcoRVR, respectively, from *C. parapsilosis* CLIB214 genomic DNA. Isolated products were fused together by PCR with primers CpNEUT5LUpupApaIStuIF and CpNEUT5LDodoStuIEcoRVR, and then digested with *Apa*I/*Eco*RV. The fragment was ligated with the *Apa*I/*Eco*RV digested pDONR^TM^ 221 backbone to create pNRVL’. A portion of pNRVL’ was amplified with pDONRnheI-SpeIF and pDONRnheI-SpeIR, digested with *Spe*I and ligated with *Nhe*I digested pNRVL’ to create pNRVL carrying no sites for *Nhe*I or *Spe*I. Plasmid pSFS2a was used as a template to amplify the nourseothricin resistance marker *caSAT1* with the primer pairs pSFS2SATEcoRIF and pSFS2SATEcoRIR (Reuss et al., 2004). The isolated amplicon was digested with *Eco*RI and ligated with *Eco*RI digested pSN40 to create pSN40-caSAT1.

To gain *LEU2* and *caSAT1* derivatives of pNRVL (pNRVL-L and pNRVL-S), *C. maltosa LEU2* with regulator sequences and dominant selection marker *caSAT1* were amplified from pSN40 and pSN40-caSAT1, respectively, with Primer5NotIF and Primer2BssHIIR, digested with the respective enzymes, and ligated with *Bss*HII/*Not*I digested pNRVL backbone.

GFP carrying derivatives of pNRVL-L and pNRVL-S were generated by releasing GFP with regulator sequences from pECpOE-GFP-L-N5L with *Xho*I/*Mlu*I digestion and the isolated fragments were then ligated into *Xho*I/*Bss*HII digested pNRVL-L and pNRVL-S to create pNRVL-L-GFP and pNRVL-S-GFP.

The reintegration of *ADE2* gene was carried out by amplifying the CPAR2_805940 ORF along with 1 kb upstream and 500 bp downstream regions with the primer pair CPAR2_805940RIXhoIF – CPAR2_805940RIMluIR from *C. parapsilosis* CLIB214 genomic DNA. The amplicon was digested with *Xho*I/*Mlu*I and ligated with *Xho*I/*Bss*HII digested pNRVL-S to gain pNRVL-S-ADE2^RI^.

### Gateway^TM^ Cloning

The GFP containing plasmid p2120 was a kind gift from Professor Judith Berman. BP and LR cloning were performed according to the manufacturer’s instructions. First, the GFP ORF was amplified with primers GFPattB1F and GFPattB2R, isolated, and then BP cloned with pDONR^TM^221 to create pENTRY-GFP. This was further LR cloned with pDCpOE-L-RP10, pDCpOE-L-N5L and pDCpOE-N-N5L to gain the respective expression clones.

### Transformation

All destination plasmids and pDONR^TM^221 were propagated in *Escherichia coli* DB3.1 strain while strain 2T1 was used to transform with all other plasmids generated. Transformation of *E. coli* strains was carried out using a standard heat-shock method. *Candida* cells were transformed using a ssDNA/LiAc/PEG mediated heat-shock method as described by Holland et al. (2014). Plasmids were digested overnight with *Stu*I according to the manufacturer’s instructions, and precipitated, then assayed on 0.8% (m/V) agarose gel to check integrity, fragment sizes and concentrations. Two μg of linear plasmids were used for transformation in respective fungal strains. Cells were plated 2 (for auxotrophy complementation) and 4 h (for dominant selectable marker) after heat-shock.

### Validation of Mutants

Colonies were subjected to rapid DNA isolation followed by PCR (primer sequences are listed in Supplementary Table S2). Amplicons were assayed on 0.8% (m/V) agarose gel (Holland et al., 2014). Positive colonies were further investigated with Southern-blot. DIG labeled probes were generated using the Roche DIG-labeling Kit with *C. parapsilosis* CLIB214 DNA according to the manufacturer’s instructions (primer sequences are listed in Supplementary Table S2). Genomic DNA isolation and Southern-blot were performed as described by Gácser et al. (2007). Ten μg of genomic DNA was digested with the appropriate restriction endonuclease, and Roche DIG-labeled DNA Molecular Weight Marker VII was used for comparison. The enzymes applied for genomic DNA digestion and expected fragment sizes are described in Supplementary Table S4.

### *In silico* Data Analysis, Editing, and Statistics

Sequences were obtained from Candida Genome Database and edited with ApE (a plasmid editor) v2.0.51 (Skrzypek et al., 2017). Alignments were made by BioEdit v7.2.6. Visualization was carried out with SnapGene Viewer v3.2.1, and pictures were edited with ImageJ v1.52h and GIMP v2.8.18. Statistical analysis was performed by GraphPad Prism 6.01. Data was evaluated using Student’s *t*-test in a combination with Mann-Whitney test. Results are presented as mean and standard deviation.

### Growth Conditions

J774.2 murine macrophage aliquots were stored in liquid nitrogen, frozen in cryoprotective medium containing (Basal Eagle’s Medium with Hanks’ balanced salt solution and 15% Dimethylsulfoxide without L-Glutamine) and were cultivated routinely in a 37°C incubator, in the presence of 5% (V/V) CO_2_ and 100% (V/V) relative humidity in Dulbecco’s modified eagle medium containing 10% (V/V) heat-inactivated Fetal bovine serum and 100 unit/ml penicillin/streptomycin solution.

*E. coli* strains were cultivated in LB media (1% (m/V) NaCl, 1% (m/V) tripone, 0.5% (m/V) yeast extract) and were maintained on LB plates supplemented with 1.5% (m/V) agar at 4°C.

*Candida* strains were kept in YPD containing 20% (V/V) glycerol at –80°C and routinely maintained on solid YPD medium (1% (m/V) glucose, 1% (m/V) peptone, 0.5% (m/V) yeast extract, 1.5% (m/V) agar) supplemented with 100 unit/ml penicillin/streptomycin solution and stored at 4°C. For dominant selectable markers YPD penicillin/streptromycin plates were supplemented with nourseothricin at a final concentration of 100 μg/ml. For auxotrophy complementation minimal media containing 0.19% (m/V) yeast nitrogen base, 2% (m/V) glucose, 1.5% (m/V), agar and 100 unit/ml penicillin/streptomycin was supplemented with drop out media (Holland et al., 2014). Selection for heterozygotes was carried out using the same media but supplemented with leucine in a final concentration of 0.5 mg/ml.

Viability of *ade2*^–/–^ and ADE2^RI^ mutants was invesitgated on minimal plates containing 0.19% (m/V) yeast nitrogen base, 2% (m/V) glucose, 1.5% (m/V) agar including 100 unit/ml penicillin/streptomycin solution without adenine or supplemented with adenine in a final concentration of 0.2 mg/ml.

Two days before the experiment strains were inoculated into 3 ml YPD liquid medium and cultivated at 30°C in an orbital shaker (150 rpm). One microliter of the overnight culture was transferred into 3 ml YPD liquid medium and incubated under the same conditions. On the day of the experiment two ml of the synchronized cultures were collected and washed twice, suspended in sterile 1x PBS, then counted with a Burker-chamber and adjusted to the proper concentration.

### Phenotypic Analysis

For the spot-assay, four step 10-fold dilution series were applied ranging from 10^4^ to 10^1^ cells/4 μl in 1x PBS. Suspensions were spotted onto the dedicated solid media representing the given condition. Applied conditions and concentrations are summarized in Supplementary Table S5. Plates were incubated for 2 days at 30 or 37°C, except for CdSO_4_ at 37°C, which was kept for 4 days.

For growth curve analysis, 150 μl of the 1.33-fold media (representing the given condition) was pipetted into 96 well plates, and then 5 × 10^5^ cells/50 μl distilled water were added. Plates were incubated at 30 and 37°C. OD_600_ was measured every other hour for 24 h.

### Fluorescent Imaging

To investigate GFP expression, the strains were cultivated overnight in YPD, washed and suspended in 1x PBS as described. Cells were either observed by using Zeiss Axio Observer 7 fluorescent microscope or by Amnis^®^ FlowSight^®^ flow cytometer and analyzed by Amnis^®^ IDEAS software.

### Association Assay

J774.2 phagocytes were plated at 1 × 10^6^/well into a 24 well plate and allowed to adhere for 1 h. Synchronized *Candida* cultures were washed two times with 1x sterile PBS and stained with Alexa Fluor 647 as described (Németh et al., 2013). Cell concentration was adjusted to reach a final host:yeast ratio of 1:5. Equal amount of sterile 1x PBS was added to the non-infected control. The experiment was performed in three biological parallels. After 1 h of co-incubation, the plate was placed on ice and cells were carefully washed and then detached by pipetting with ice cold 1x PBS. Cell suspensions were immediately measured by Amnis^®^ FlowSight^®^ instrument and evaluated by using Amnis^®^ IDEAS software.

### Cytotoxicity-Assay [Measurement of Lactate Dehydrogenase (LDH) Activity]

J774.2 cells were plated at 1.5 × 10^5^/well into a 96-well plate and allowed to adhere for 1 h. Yeast cells were added to a host:yeast ratio of 1:5, equal amount of sterile PBS was applied for negative and positive control. The experiment was performed in three biological parallels. After 24 h of incubation, 0.1% (m/V) Triton-X 100 was added into positive control wells to disrupt the cells and determine maximum LDH release. LDH was measured using Takara LDH Cytotoxicity Detection Kit according to the manufacturer’s instructions. Data were analyzed by subtracting the non-infected control values from infected and positive control samples, and then correlated to the maximum LDH release (positive control) to determine cytotoxicity percentage.

### Deletion and Reintegration of *ADE2* (CPAR2_805940)

The CPAR2_805940 knock-out mutant was generated according to the double auxotrophy complementation method developed by Holland et al. (2014). Briefly, *C. maltosa LEU2* and *C. dubliniensis HIS1* markers were amplified from pSN40 and pSN52, respectively, with primers Primer_2 and 805940_Primer_5 (Noble and Johnson, 2005; Holland et al., 2014). Approximately 500 bp regions lying upstream and downstream from CPAR2_805940 ORF were amplified with the primer pairs 805940_Primer_1 – 805940_Primer_3 and 805940_Primer_4 – 805940_Primer_6. The PCR products were stitched with *CmLEU2* or *CdHIS1* amplicons by fusion PCR with primers 805940_Primer_1 – 805940_Primer_6 to create deletion cassettes that were used to sequentially transform the *his1^–/–^*/*leu2^–/–^* double auxotroph strain CPL2H1 to gain *ade2^–/–^.*

## Results

### Introduction of an Integrative Overexpression Gateway^TM^ Plasmid Targeting *C. parapsilosis CpRP10*

The most preferred locus for knock-in approaches in *C. albicans* is *RPS1*, also known as *RP10* or orf19.3002 (encodes the ortholog of the ribosomal protein 10 from *Saccharomyces cerevisiae*), because constructs integrated into this region provide strong, homogenous expression that have been exploited in many studies (Swoboda et al., 1995; Chauvel et al., 2012; Legrand et al., 2018; Znaidi et al., 2018). In agreement with this, we identified the ortholog of *RPS1* in *C. parapsilosis* (CPAR2_110290) using the BLAST search function of the Candida Genome Database and named it *CpRP10* (Skrzypek et al., 2017). By sequential modification of pCA-DEST1300 (a Gateway^TM^ compatible derivative of CIp10 carrying a constitutive TDH3 promoter from *C. albicans* and *URA3* selection marker besides the *RPS1* target locus) designed for overexpression (OE) purposes, we generated a pDCpOE-L-RP10 destination plasmid by replacing the *URA3* marker to *LEU2* and the *RPS1* target to *CpRP10*, thereby making it compatible with a leucine auxotroph *C. parapsilosis* (CPL2) strain (Murad et al., 2000; Chauvel et al., 2012; Holland et al., 2014; Legrand et al., 2018). This plasmid served as an acceptor in the LR cloning reaction with pENTRY_GFP to create GFP containing pECpOE-GFP-L-RP10, which, after linearization at the artificially introduced *Stu*I site in the middle of *CpRP10*, could be used to transform the leucine auxotroph laboratory strain.

### Expression Construct Integrated Into the CpRP10 Locus Lead to Heterogenous Expression

**FIGURE 1:**
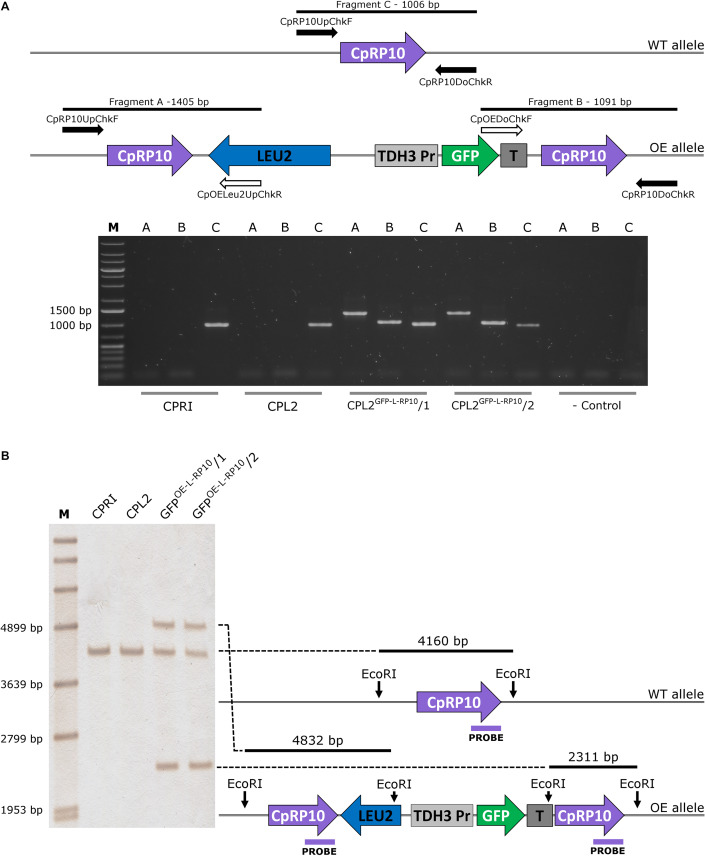
Validation of the GFP-expressing transformants by PCR **(A)** and Southern-blot **(B)**. **(A)** The solid arrows represent primers specific for genomic DNA, and empty arrows show primers specific for plasmid sequences. PCR reaction A and B yield amplicons only when the integration is correct, and validate the overexpression construct. Fragment C is present in both cases, as integration affects only one of the two alleles. **(B)** Represents the scheme for validation using Southern-blot. CPRI served as a control for the experiments, CPL2 is the parental strain of CPL2^GFP–L–RP10^/1 and /2 GFP-expressing transformants.

**FIGURE 2:**
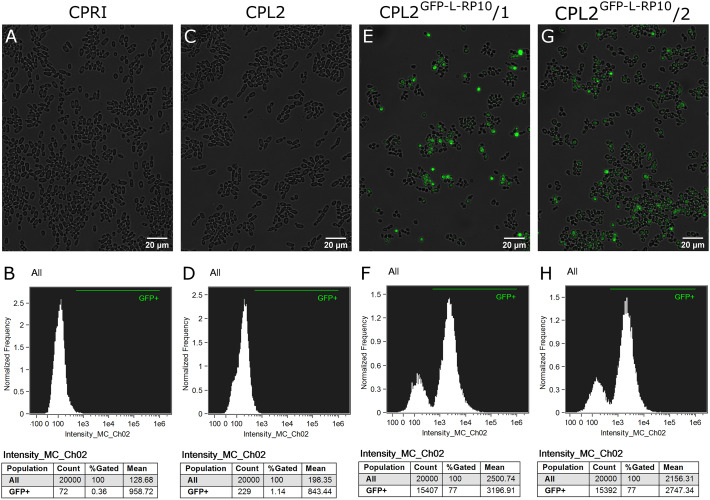
Characterization of the GFP-expressing mutants by fluorescent imaging. **(A,C,E,G)** Show fluorescence microscopic images while panel **(B,D,F,H)** represent the statistical analyses of the GFP expression by flow cytometer. CPRI served as a control for the experiments, CPL2 is the parental strain of CPL2^GFP–L–RP10^/1 and /2 GFP-expressing transformants.

The integration of linearized pECpOE-GFP-L-RP10 in single cell colonies derived from two independent mutants were analyzed by colony PCR and Southern-blot, and positive candidates were sequenced to further examination (Figure 1). In addition, the GFP-expressing transformants, CPL2 and its *Candida maltosa LEU2* complemented derivative (CPRI) were included (Holland et al., 2014). Interestingly, when validated mutants were investigated with a fluorescent microscope, a heterogenous GFP expression was observed in the population. A small portion of cells emitted strong fluorescent green light, the majority of cells emitted weak GFP signal, and some cells did not fluoresce. This phenomenon was further examined and statistically evaluated by Amnis^®^ Flowsight^®^ equipment using the two independent transformants with the respective control strains (Figure 2). We repeated the transformation procedure two more times and generated single cell colonies by collecting single cells from the top of solid media and re-plating them three consecutive times but the heterogenous expression persisted in the population.

### CpNEUT5L Locus Provides Strong, Homogenous GFP Expression in *C. parapsilosis*

**FIGURE 3:**
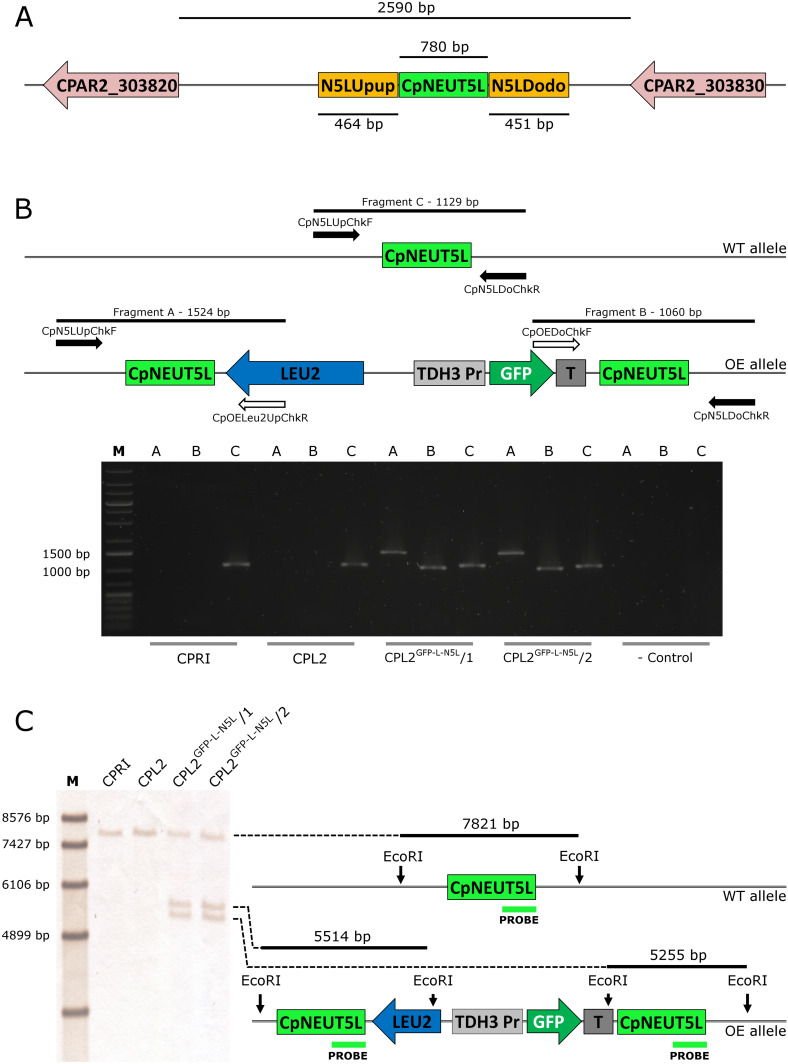
**(A)** Localization of the CpNEUT5L (N5L) locus. N5L is a 780 bp long sequence mapping onto a 2,590 bp intergenic region between CPAR2_303820 and CPAR2_303830, and it is the target of the pECpOE-N5L integrative plasmids. Up- and downstream flanking sequences of N5L (called N5LUpup and N5LDodo respectively) are target sites for the pNRVL vector. **(B)** Shows the validation of the GFP-expressing mutants by PCR. Solid arrows represent primers specific for genomic DNA and empty arrows show primers specific for plasmid sequences. PCR reaction A and B yield amplicons only when the integration is correct, and validate the overexpression construct. Fragment C is present in both cases, as integration affects only one of the two alleles. **(C)** Is a schematic representation of the validation using Southern-blot. CPRI served as a control for the experiments, CPL2 is the parental strain of CPL2^GFP–L–N5L^/1 and /2 GFP-expressing transformants. Note that scheme is not drawn to scale.

As GFP expression was present, we concluded that the promoter is capable of inducing GFP expression, which suggested that the *CpRP10* locus might be the factor leading to a heterogenous population in terms of GFP expression. With this in mind, we decided to alter the target region to the ortholog of the intergenic region NEUT5L of *C. albicans*. This is a 550 bp long sequence lying between orf19.1963 and orf19.1961 on a 3,789 bp long intergenic region on the left arm of chromosome five. It has been utilized as an alternative locus to integrate different constructs instead of using *RPS1*. Importantly, *C. albicans* strains hemizygous to NEUT5L do not show any growth defect or alterations in the filamentous growth, which makes NEUT5L an ideal sequence to target in *C. albicans* (Gerami-Nejad et al., 2013). BLAST search revealed that the NEUT5L ortholog in *C. parapsilosis* is located between CPAR2_303820 and CPAR2_303830 (Skrzypek et al., 2017). These two genes lie 2,590 bp from each other and we selected a 780 bp long sequence in the region that we named CpNEUT5L (N5L), referring to the homology rather than localization (Figure 3A). Accordingly, we altered the pDCpOE-L-RP10 plasmid by replacing the target locus CpRP10 to N5L to create pDCpOE-L-N5L, which was used to gain its GFP carrying derivative (pECpOE-GFP-L-N5L) by LR cloning. After transformation single cell colonies were generated from two independent prototroph colonies and verified by PCR and Southern-blot (Figures 3B,C).

**FIGURE 4:**
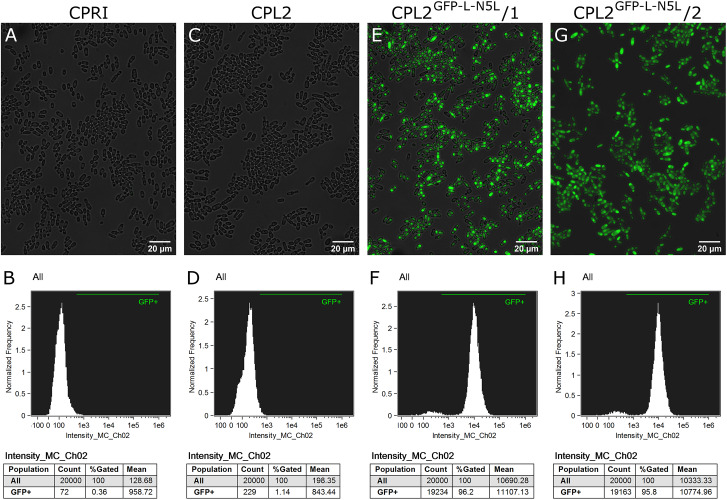
Characterization of the GFP-expressing mutants by fluorescence imaging. To generate fluorescently tagged strains, the pECpOE-GFP-L-N5L construct was applied. **(A,C,E,G)** show fluorescence microscopic images while panel **(B,D,F,H)** represent the statistical analyses of the GFP expression by flow cytometer. CPRI served as a control for the experiments, CPL2 is the parental strain of CPL2^GFP–L–N5L^/1 and /2 GFP-expressing transformants. Note, that the histogram of controls shown here **(B,D)** are the same as ones presented in Figures 2B,D.

The validated strains were subsequently analyzed by fluorescence microscopy and Amnis^®^ Flowsight^®^ flow cytometer. According to our observation, 95% of the total population emitted a strong GFP signal, suggesting that N5L but not *CpRP10* provides strong, homogenous expression (Figure 4).

### Application of pDCpOE-N5L in Clinical Isolates

To obtain GFP-stained prototroph *C. parapsilosis* strains, we modified pDCpOE-L-N5L for use with clinical isolates. The auxotrophy marker was replaced to the codon optimized version of the dominant selectable marker nourseothricin acetyl transferase 1 (*caNAT1*), which neutralizes the antifungal agent nourseothricin (NTC) (Gerami-Nejad et al., 2012). The plasmid pECpOE-GFP-N-N5L was used to generate GFP-expressing derivatives from a total of six *C. parapsilosis* isolates (CDC317, CLIB214, CBS 1954, CBS 2211, CBS 6318, GA1) (Nosek et al., 2002; Laffey and Butler, 2005; Gácser et al., 2007; Butler et al., 2009). Candidates were verified by molecular methods and analyzed by Amnis^®^ Flowsight^®^ flow cytometer (Supplementary Figures S1, S2). We established that more than 99% of the total population emitted green fluorescent light independent of the parental strain (Supplementary Figures S3, S4).

### Developing CpNEUT5L Targeting Replacement Cassette

**FIGURE 5:**
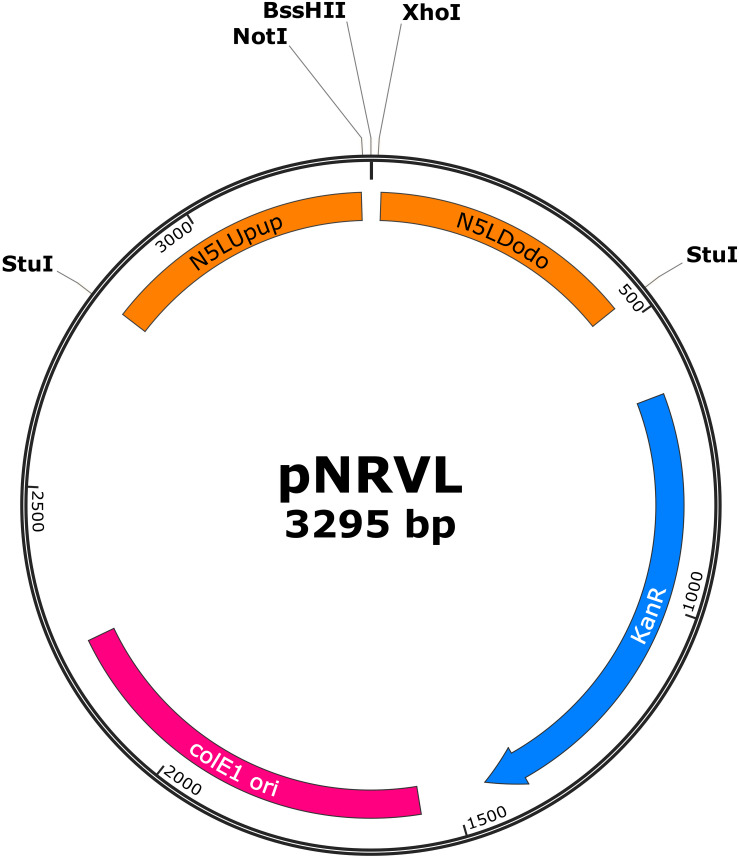
Map of pNRVL, a vector encoding a replacement cassette targeting CpN5LUpup and CpN5LDodo. Selection markers and gene of interest can be cloned between *Not*I/*Bss*HII and *Bss*HII/*Xho*I, respectively. Replacement cassette can be released by *Stu*I digestion.

**FIGURE 6:**
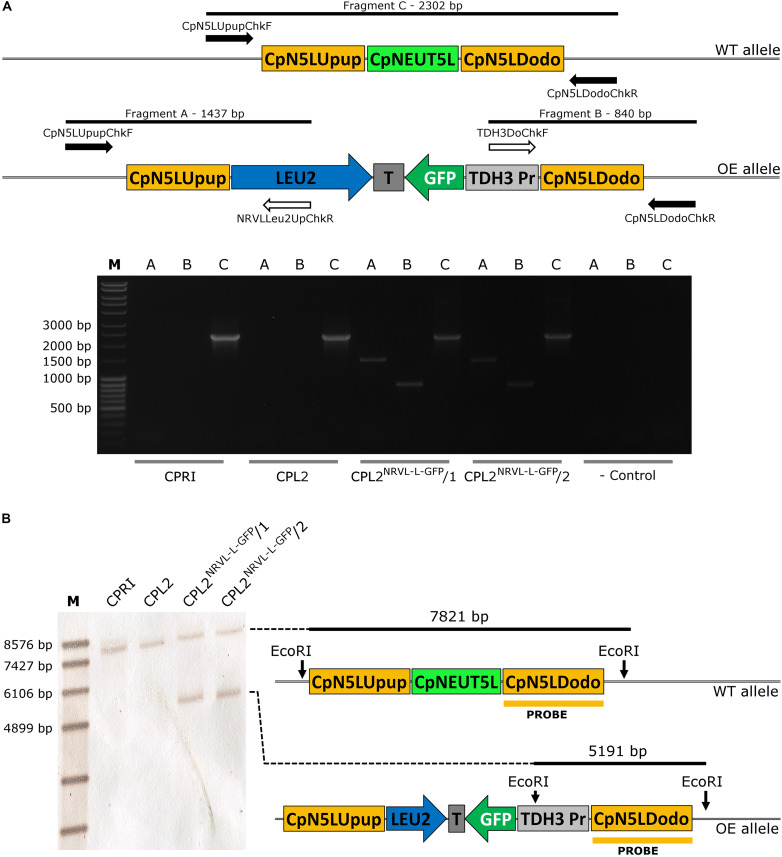
Validation of the GFP-expressing transformants carrying pNRVL-L-GFP by molecular techniques. **(A)** Describes the validation with primers specific to the genomic DNA (solid arrows) or to the plasmid sequence (empty arrows). **(B)** Presents the conception of validation using Southern-blot. CPRI served as a control for the experiments, CPL2 is the parental strain of CPL2^NRVL–L–GFP^/1 and /2 GFP-expressing transformants.

**FIGURE 7:**
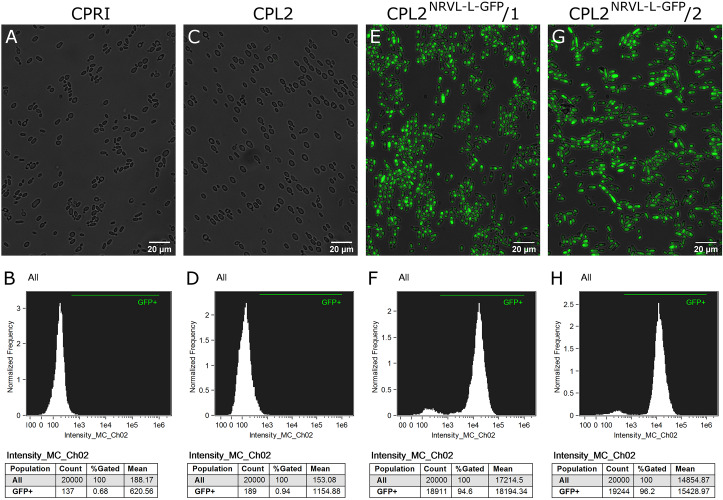
Characterization of the GFP-expressing mutants by fluorescence imaging. Strains were generated with the replacement cassette released from pNRVL-L-GFP. **(A,C,E,G)** Show microscopic images while panel **(B,D,F,H)** represent statistical analysis of the GFP expression by flow cytometer. CPRI served as a control for the experiments, CPL2 is the parental strain of CPL2^NRVL–L–GFP^/1 and /2 GFP-expressing transformants.

For mutant generation, in general, we observed a transformation efficiency of <5 transformants/μg DNA. We suspected that this poor outcome could have been due to the relatively large size of the transforming DNA (7764 bp for pECpOE-GFP-L-N5L and 6753 bp for pECpOE-GFP-N-N5L) as the constructs used for *Candida* transformation also contain irrelevant sequences in the fungus such as colE1 replication origin and selection marker for plasmid propagation. The other drawback of using an integrative plasmid is that it duplicates the locus it targets. Once such recombination occurs in a diploid organism, the target locus will be present in three copies, one on the unaffected allele and two flanking the integrated construct on the other. This latter, without continuous selection pressure, might lead to the loss of the integrated sequence via spontaneous intrachromosomal recombination. This is a well known event in *C. albicans*, which occurs randomly by nature leading to chromosomal rearrangement and elimination of endogenous sequences, but it is also purposefully exploited for targeted gene alteration, such as by the URA-blaster method or the recently introduced CRISPR/Cas9 approach published by Fonzi and Irwin (1993); Todd et al. (2019), and Nguyen et al., 2017. Moreover, upon subsequent transformation with a similar integrative plasmid, there are three potential target sites where it can randomly integrate. This might lead to unwanted genomic diversity from strain to strain, which could affect the phenotype of the transformants and hamper their consistent analysis. To circumvent these issues, we designed a classical replacement cassette targeting the up- and downstream flanking sites of N5L (we named them CpNEUT5L Upup and Downdown – N5LUpup and N5LDodo, respectively) (Figure 3A). Therefore, removing one of the N5L loci by the replacement cassette first, there will be only one locus to target with one of the integrative Gateway^TM^ plasmids. This approach leads to a more unified set of mutants in terms of genetic background when cumulative knock-in procedures are required. To achieve this, we introduced pNRVL, which was constructed by manipulating the Gateway^TM^ plasmid pDONR^TM^221. The attP1 and attP2 sites and the two genes in between were removed, one of them is responsible for chloramphenicol resistance and the other, ccdB, encodes a girase inhibitor that provides negative selection during pENTRY generation in a compatible *E. coli* strain. pNRVL carries up and downstream flanking sequences of N5L with dedicated restriction sites (*Not*I, *Bss*HII, and *Xho*I) in the middle and flanked by *Stu*I sites for release (Figure 5). We first equipped pNRVL with the selection marker *C. maltosa LEU2* from pSN40 (Noble and Johnson, 2005; Holland et al., 2014). It is directly compatible with any constructs created with the previously described Gateway^TM^ system as genes cloned into pECpOE-N5L can be released by *Xho*I/*Mlu*I digestion together with P_CaTDH__3_ and the terminator (*Mlu*I and *Bss*HII provide compatible ends). The cassette for transformation can be released by *Stu*I digestion. To test this vector, we utilized pNRVL-LEU2 (pNRVL-L) carrying P_CaTDH__3_-GFP-TER_ScURA__3_ fragment (pNRVL-L-GFP) and transformed the CPL2 strain. Two independent colonies were collected and analyzed in deeper detail by molecular techniques and fluorescent imaging as detailed above (Figure 6). By using Amnis^®^ Flowsight^®^, we established that the GFP signal was under 1% in the population of CPL2 parental and CPRI control strains, while it was over 94% in GFP-labeled transformants (Figure 7).

### Adaptation of pNRVL for Utilization in Prototroph Strains

To make pNRVL compatible with clinical isolates, we applied the codon optimized version of the dominant selectable marker streptothricin acetyltransferase *caSAT1* from pSFS2a to gain pNRVL-caSAT1 (pNRVL-S) (Reuss et al., 2004). It was integrated into the fusion PCR based deletion cassette construction workflow by replacing *CmLEU2* to *caSAT1* on plasmid pSN40 (pSN40-caSAT1); therefore, similarly to the *LEU2* marker, it can also be amplified using primer pairs Primer5NotIF, Primer2BssHIIR (see section “Materials and Methods”) (Holland et al., 2014). As before, we modified six prototroph *C. parapsilosis* strains previously mentioned with *Stu*I linearized pNRVL-S-GFP to generate their GFP-expressing derivatives. Single cell colonies were collected, verified by using molecular techniques and analyzed by Amnis^®^ FlowSight^®^ (Supplementary Figures S5, S6). While the GFP positive portion of the parental strains ranged from 0.04 to 1.12%, over 99% of the mutant populations were emitting green fluorescent light (Supplementary Figures S3, S4). With observations regarding expression mutants gained by using either pECpOE-GFP-L/N-N5L or pNRVL-L/S-GFP, we found that the larger population of cells was categorized as GFP positive by flow cytometer when *caNAT1*/*caSAT1* marker rather than *LEU2* were applied independently of the construct (pECpOE or pNRVL) used. Statistical analysis revealed significant differences (*p* = 0.0011) with LEU2 constructs (95.7 ± 0.7572) in comparison with dominant selectable markers (99.26 ± 0.2392) (Supplementary Figure S7).

### Alteration of CpNEUT5L Does Not Affect the Growth Rate Under Different Stress Conditions and the Fundamental Virulence Properties of *C. parapsilosis*

We investigated whether alteration of N5L locus had any effect on the basic physiological properties of the fungus. To achieve this we exposed the GFP-expressing mutants carrying either pECpOE-L-GFP or pNRVL-L-GFP to 20 different conditions and compared their fitness to that of CPRI and CPL2 control strains. Conditions included pH = 4–8, oxidative (CdSO_4_, CuCl_2_, CuSO_4_, H_2_O_2_) -, osmotic (Glycerol, NaCl, Sorbitol) – and cell-wall (Caffeine, Calcofluor white, Congo red) stressors, bivalent cation starvation (EDTA), and membrane disturbance (SDS), and we also examined their viability in/on minimal mediums (YCB and YNB) and protein utilization (see Supplementary Table S5). Growth capability was monitored by cultivating the cells in liquid (growth curve) as well as on solid media (spot assay). No alteration in the growth or difference in the colony morphology was found under any of the conditions we applied showing that the integration of expressing constructs into this intergenic region does not affect the fitness of *C. parapsilosis* (Supplementary Figures S8–S15).

**FIGURE 8:**
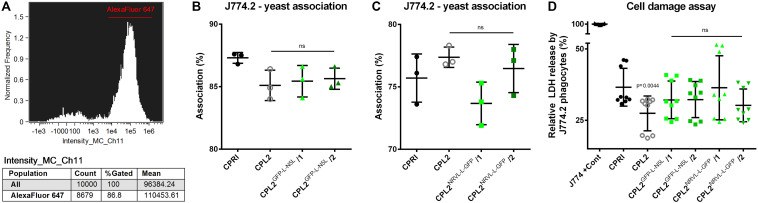
Characterization of the virulence properties of the GFP-expressing strains. Alexa Fluor 647 labeled fungi were co-incubated with J774.2 murine phagocytes with a host:pathogen ration of 1:5. After 1 h of incubation, the portion of macrophages associated with yeast cells relative to the total number of J774.2 was determined. **(A)** Shows a representative histogram. **(B,C)** Describe the percentages of the Alexa Fluor 647 positive J774.2 population. LDH release from the macrophages was determined after 24 h of incubation with the dedicated fungal strains. **(D)** Summarizes the relative LDH released normalized to the total LDH content of the macrophages. CPRI served as a control for the experiments, CPL2 is the parental strain of CPL2^GFP–L–N5L^/1,/2 and CPL2^NRVL–L–GFP^/1,/2 GFP-expressing transformants. Experiments were performed twice in triplicates. Statistical analysis was performed by Student’s *t*-test with Mann-Whitney test.

To test the basic virulence attributes of the expression mutants, we co-incubated the GFP-expressing transformants and CPL2, CPRI strains with the cells of the J774.2 murine macrophage like cell-line. We first determined the portion of J774.2 cells associated (attached to the surface of or phagocytosed by the macrophages) with fungal cells. We applied Alexa Fluor 647 succinimidyl ester stained yeasts (emitting red fluorescent light) and co-incubated them with the host cells for 1 h (host:pathogen ratio of 1:5). The extent of association was determined by Amnis^®^ FlowSight^®^ equipment as the ratio of Alexa Fluor 647 positive macropohage population compared to the total number of J774.2 cells (Figure 8A).

According to these measurements, no significant differences were found between CPRI and any other mutant. When macrophages were incubated with CPL2^GFP–L–N5L^ strains, association percentages were 87.3 ± 0.4359% (CPRI), 85.1 ± 1.212% (CPL2), 85.43 ± 1.258 and 85.63 ± 0.8505% (CPL2^GFP–L–N5L^/1 and /2 respectively) (Figure 8B). In the case of pNRVL derivatives, these values were 75.5 ± 1.931 + (CPRI), 77.37 ± 0.8145% (CPL2) 73.67 ± 1.714% and 76.47 ± 1.930% (CPL2^NRVL–L–GFP^/1 and /2 respectively) (Figure 8C).

Host damage caused by the fungal cells during the interaction was also investigated. This was carried out by the measurement of lactate-dehydrogenase (LDH) in the supernatant. LDH is an intracellular enzyme that is released only when the cell membrane loses integrity; therefore, a higher LDH activity equates to greater cell damage. Unstained fungal strains were co-incubated with J774.2 cells for 24 h (host:pathogen ratio of 1:5). Relative cell damage was calculated by normalizing the data to the maximum LDH content of the cells (Figure 8D). Significant difference in the damage capacity (*p* = 0.0019) was observed only in the comparison of CPRI (36.59 ± 6.637%) with CPL2 (27.44 ± 6.105%). Although the experiment was performed in a complete media, the results indicate that it is still possible that the auxotrophy affects the phagocyte damaging capacity of this mutant. LDH release caused by the GFP-expressing mutants were 32.16 ± 6.587%, 32.26 ± 6.326%, 36.40 ± 11.25%, and 30.24 ± 5.733% (CPL2^GFP–L–N5L^/1,/2,CPL2^NRVL–L–GFP^/1 and /2, respectively) that were not significantly different from the one of CPRI.

### Wildtype *ADE2* Allele Integrated Into CpNEUT5L Complements *ade2^–/–^* Phenotype

**FIGURE 9:**
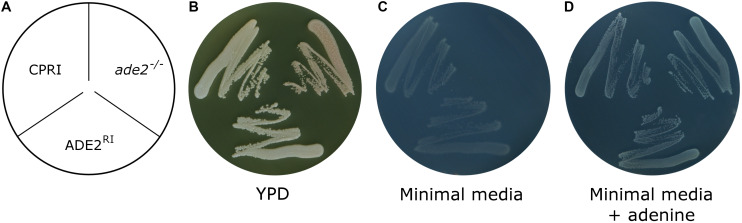
Growth assay of *ade2^– /–^* and ADE2^RI^ mutants. **(A)** Shows the layout of the strains. Growth was investigated by plating the cells on YPD complete media **(B)** or minimal media supplemented with **(C)** or without adenine **(D)** (see section “Materials and Methods”). CPRI was included as a control. YPD plate was incubated for 3 days, plates with minimal media supplemented with or without adenine was incubated for 8 days at 30°C.

Gene function analysis is most commonly carried out by knocking out the gene in question. To make sure that the potential phenotypic alteration is a consequence of the loss of the gene (and its product) and not due to unwanted ectopic integration, generation of a reintegrant strain is mandatory. Integrating the given ORF into the native locus requires multiple cloning steps, which is time consuming. As we needed a rapid workflow to generate the reintegrant derivatives of our knock-out mutant collection, we examined whether pNRVL is suitable for that purpose. As a proof of principle, we deleted both alleles of *ADE2* gene by the double auxotrophy complementation method (Holland et al., 2014; Tóth et al., 2018). The lack of *ADE2* gene results in an obvious phenotype, since the precursor of the Ade2 enzyme is accumulated in the cells turning colonies into red. This mutant has its histidine and leucin auxotrophy complemented, but obviously it could grow only in the presence of adenine. To generate the reintegrant strain, *ADE2* ORF with 1 kbp upstream and 500 bp donwstream regulator sequences were amplified and cloned into pNRVL-S to create pNRVL-S-ADE2^RI^, which was used to transform *ade2^–/–^*. Deletion and reintegrant mutants were verified using PCR and Southern-blot (Supplementary Figures S16, S17 respectively). Their growth was observed on complete and minimal media supplemented with or without adenine (Figure 9). All strains could grow on complete media with CPRI and ADE2^RI^ colonies showing the same white color, while the *ade2^–/–^* mutant was red. All strains could grow on minimal media supplemented with adenine, but only CPRI and ADE2^RI^ formed colonies on the minimal media without adenine. This indicates that only a single copy of *ADE2* gene inserted into N5L can complement the mutant phenotype.

## Discussion

*C. parapsilosis* is a significant neonatal pathogen, yet we still know very little about its pathogenicity and physiological aspects (Pammi et al., 2013). In part, the reason for this is that the number of the molecular tools available that lags behind those existing for *C. albicans*. However, although several knock-out techniques have been developed to date, no locus has been described as an acceptor sequence for knock-in approaches in *C. parapsilosis* (Ding and Butler, 2007; Gácser et al., 2007; Holland et al., 2014; Gonia et al., 2017). The goal of this study was to identify and characterize a genomic region for integrating ectopic DNA in *C. parapsilosis* and develop compatible plasmids.

We first analyzed *CpRP10*, the ortholog of *RPS1* in *C. albicans*, as it is one of the most preferred loci for integrating exogenous DNA in that species (Chauvel et al., 2012; Gerami-Nejad et al., 2013). We adopted an integrative plasmid designed for overexpression that takes advantage of the Gateway^TM^ cloning method (Znaidi et al., 2018). We generated a GFP-expressing derivative utilizing the *LEU2* gene for selection. Surprisingly, the cell population arisen from single cell colonies was heterogenous in terms of GFP expression, as only approximately 75% of the total population was GFP positive. This problem was eventually solved by replacing the target locus to the ortholog of the intergenic locus NEUT5L of *C. albicans* (Gerami-Nejad et al., 2013). The same expression plasmid integrated into N5L provided homogenous green fluorescence. The necessity of generating GFP-expressing mutants of prototroph strains required the replacement of the auxotrophic marker to the dominant selectable marker *caNAT1*. This expression construct can be used in diverse NTC sensitive strains as demonstrated by applying it in six different wild-type isolates by creating their respective GFP positive derivatives. This makes the fluorescent dying procedure unnecessary, which reduces the time required for preparation, for instance, before virulence studies, and also enables longer incubation times, since cytoplasmic GFP signal, in contrast to cell-wall fluorescent dyes, does not get attenuated from generation to generation in the population. Moreover, enabling the Gateway^TM^ system in *C. parapsilosis*, provides the opportunity to facilitate the generation of gene overexpression mutants in large scale. This could represent an alternative approach for gene function analysis and could complement our results gained from the characterization of our knock-out mutant collection (Tóth et al., 2018).

In addition to the integrative plasmid, we also generated a classical replacement cassette, pNRVL, that targets the up- and downstream flanking sequences of N5L, which, therefore, eliminates N5L on either of the alleles. The intact N5L allele can serve as an acceptor sequence for the integrative plasmid if another construct needs to be introduced. A replacement cassette has an additional advantage over an integrative plasmid, as the target sequence will not be duplicated. Duplication of the target sequence results in the generation of repetitive sequences that might lead to unwanted loss of the integrated construct in the absence of selection (Todd et al., 2019). Similar to pDCpOE, pNRVL is also equipped with an auxotrophic and a dominant selectable marker. This latter provides resistance to NTC, but the protein itself is different. In the case of pDCpOE we applied *caNAT1*, but after transformation, we could observe several small colonies amongst the large ones. Most of the large colonies were confirmed by molecular methods in terms of the correct integration, but we did not know why the small colonies appear. Gonia et al. (2017) reported that the transformation procedure represents a serious stress to the cells, that could be an explanation for this phenomenon. This might affect the initial growth rate of the transformants after plating that might result in smaller colonies by the end of the incubation period. Emergence of spontaneous resistance to NTC represents an alternative explanation. It is also possible that they are clones with ectopic integration in which the expression of the selection marker is downregulated due to the genomic region, or their expression is leaky and provides only partial resistance resulting in reduced growth. This phenomenon was not investigated in more detail, however, as it was not the purpose of this present study. Considering that this issue might be due to the selection marker, we applied *caSAT1* (that also is used for selection against NTC) rather than *caNAT1* on pNRVL, but this alteration did not solve the problem either as small colonies still arose after transformation. This phenomenon might be disturbing, but we found that large colonies more likely carry the constructs integrated in the correct way; thus, we suggest collecting only the largest ones. During our work we applied only GFP as a fluorescent protein and *LEU2*/*caSAT1* for selection, but it is likely that there is no limitation on integrating more selection markers into the workflow, and also to include more fluorescent proteins (CFP, YFP, mCherry, RFP). This also enables the performance of competition assays if required, and can maximize the compatibility with fluorescent imaging techniques. By reducing the size of the transforming DNA by 2659 and 1982 bp (pNRVL-L-GFP compared to pECpOE-GFP-L-N5L and pNRVL-S-GFP compared to pCpOE-GFP-N-N5L respectively), we achieved an increase in the transformation efficiency to approximately 10–20 colonies/μg DNA.

When fungi were co-incubated with J774.2 macrophages the association percentages of the control strains (CPRI and CPL2) were different in the two experiments (Figures 8B,C). In the case of RAW264.7 cell line (an alternative murine phagocyte cell line), it has been established that prolonged passages might affect the reliability of the results as specific attributes of the population change over time (Taciak et al., 2018). Slightly similar phenomenon was established with J774 phagocytes in terms of activation by mechanical stimulation that can happen even upon the passaging procedure (Lam et al., 2009). This eventually might affect gene expression and the overall behavior of the cells. We utilize J774.2 macrophages only for 8 weeks (approximately 20 passages) after they have been thawed because their character changes over time, which might reflect an alteration of their phagocytic capacities as well. Experiments involving CPL2^GFP–L–N5L^ and CPL2^NRVL–L–GFP^ mutant pairs were not performed at the same time, which, taking the literature into account, could explain this phenomenon. Although the control strains do not match exactly in the two experiments, regarding the characterization of the GFP-expressing mutants, these results are still valid since association percentages of the GFP-expressing mutants were compared to the corresponding controls.

In addition to fluorescent tagging of a strain, pNRVL also provides an obvious way to perform gene reintegration. Reintegration of an eliminated gene into its native locus is time consuming in general due to multiple cloning steps. Significant time can be saved by dedicating one locus to accept reintegrant constructs as the generation for this requires only one ligation. We verified that, in the case of *ADE2*, one copy of the gene with its native regulatory elements integrated into N5L restored the wild-type phenotype. It is known, however, that in some cases reintroduction of just one of the two deleted alleles might be insufficient as gene dosage effect can play a significant role in *Candida* species (De Bernardis et al., 1998; Almeida et al., 2008; Zavrel et al., 2012). Since a second dominant selectable marker is not yet available in *C. parapsilosis*, the reintegration of both alleles is not currently possible in a prototroph strain. According to this, N5L itself does not solve the problem of dose dependency, but the artificial overexpression of a single copy of the gene might overcome this issue and could still complement the mutant phenotype. Overexpression can be achieved easily, as it requires only the replacement of the GFP ORF to the given ORF on pNRVL-S-GFP, which can be achieved by *Cla*I/*Nhe*I digestion followed by ligation. The effect of overexpression on the dose dependency, however, has not been tested and it probably depends on the specific gene in question.

When one would like to integrate an ectopic DNA and perform phenotypic analysis or virulence studies with a mutant, it is important to be sure of that the alteration itself does not have any effect on the examined parameters. To characterize the fitness of the GFP-expressing transformants, we investigated their viability under 20 different stress conditions by cultivating them in liquid and on solid media. Additionally, we also examined basic virulence properties of the transformants carrying the expression constructs. Phenotypic characterization and virulence studies of the GFP-tagged mutants revealed no difference in the viability or the growth rate of the transformants compared to the control strains, and they also did not differ in their ability to resist to phagocytosis or damage J774.2 macrophages. As we didn’t test all possible conditions, relevant controls for each generated transformant should be undertaken to verify expected results. This includes CPRI or the given wild-type isolate and, more importantly, especially for overexpression purposes, an irrelevant gene placed under the regulation of the same promoter. In our case this might be represented by strains generated with pECpOE-GFP-L/N-N5L or pNRVL-L/S-GFP constructs.

Taken together, this study on the N5L region represents the first detailed report of a locus for knock-in approaches in *C. parapsilosis*, which, in combination with the generated plasmids, significantly expands the existing set of molecular tools available for genetic manipulation and further facilitates the characterization of the properties of this pathogenic yeast.

## Data Availability Statement

All strains and plasmids generated in this study are available upon request. Plasmid sequences are uploaded to GanBank. Accession numbers can be found in Supplementary Table S3.

## Author Contributions

AG and TN designed the study. The original draft was prepared by TN. Reviewing and editing was managed by AG and CV. Plasmid editing was carried out by TC and TN. CP performed fluorescent imaging, all other experiment was performed by TN.

## Conflict of Interest

The authors declare that the research was conducted in the absence of any commercial or financial relationships that could be construed as a potential conflict of interest.
