# Supplementary material for: Identification and Characterization of a Neutral Locus for Knock-in Purposes in C. parapsilosis
Source: Front Microbiol. 2020 Jun 5;11:1194. doi: 10.3389/fmicb.2020.01194 (PMC7289963; doi:10.3389/fmicb.2020.01194)

Supplementary Material

**Supplemetary Figure S1.** Validation of the GFP-expressing transformants generated with pECpOE-GFP-N-N5L by PCR. **(A)** shows the conception of the validation with primers specific to the genomic DNA (solid arrows) or to the plasmid sequence (empty arrows). GFP-labeled derivatives of CDC317, CLIB214 **(B)**, CBS 1954, CBS 2211 **(C)** and CBS 6318, GA1 **(D)** were generated.


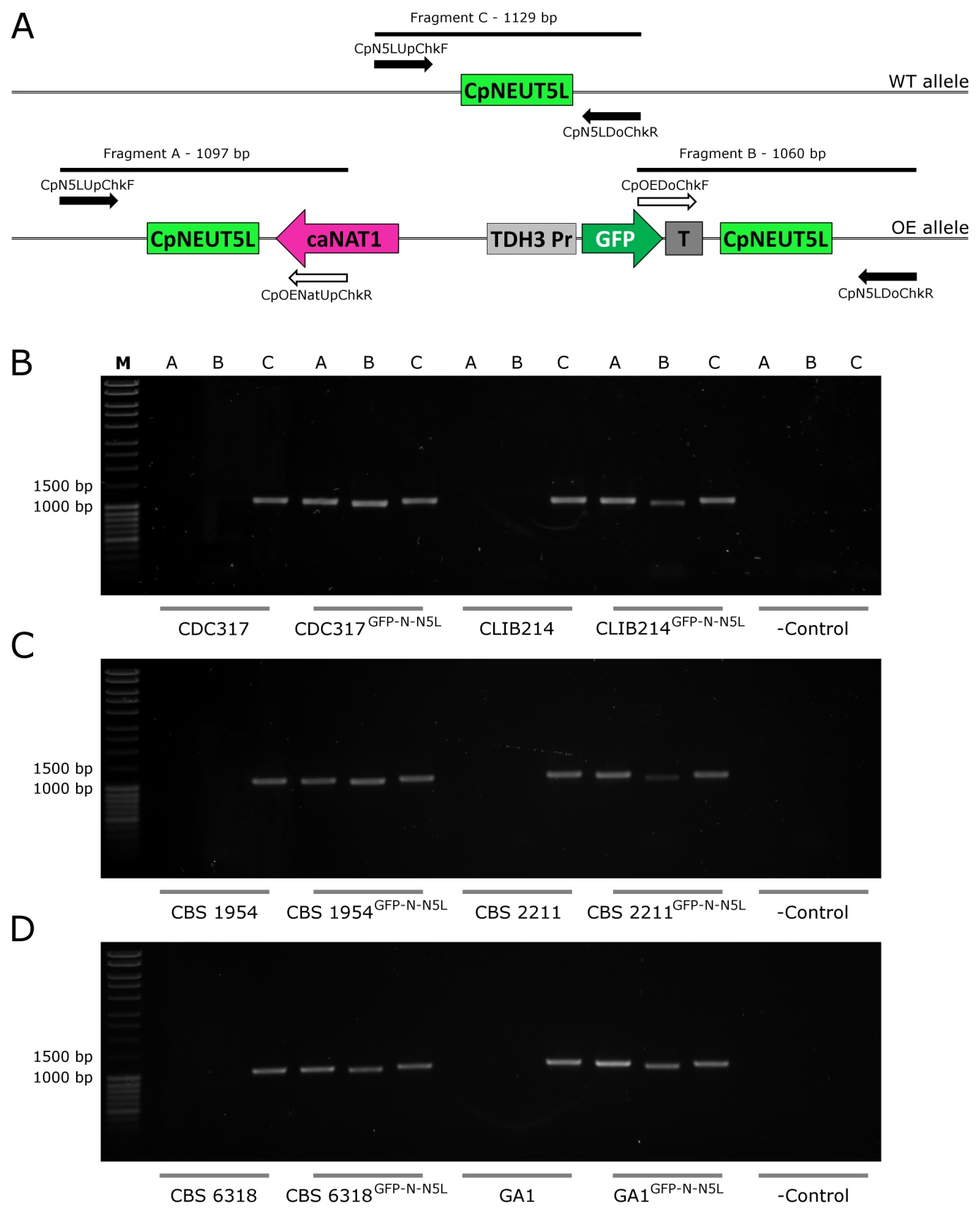


**Supplementary Figure S2.** Validation of the GFP-expressing transformants by Southern-blot. Six prototroph *C. parapsilosis* isolates were GFP-labeled by transformation with pECpOE-GFP-L-N5L. Note that scheme explaining the concept of the validation is not drawn to scale.

**
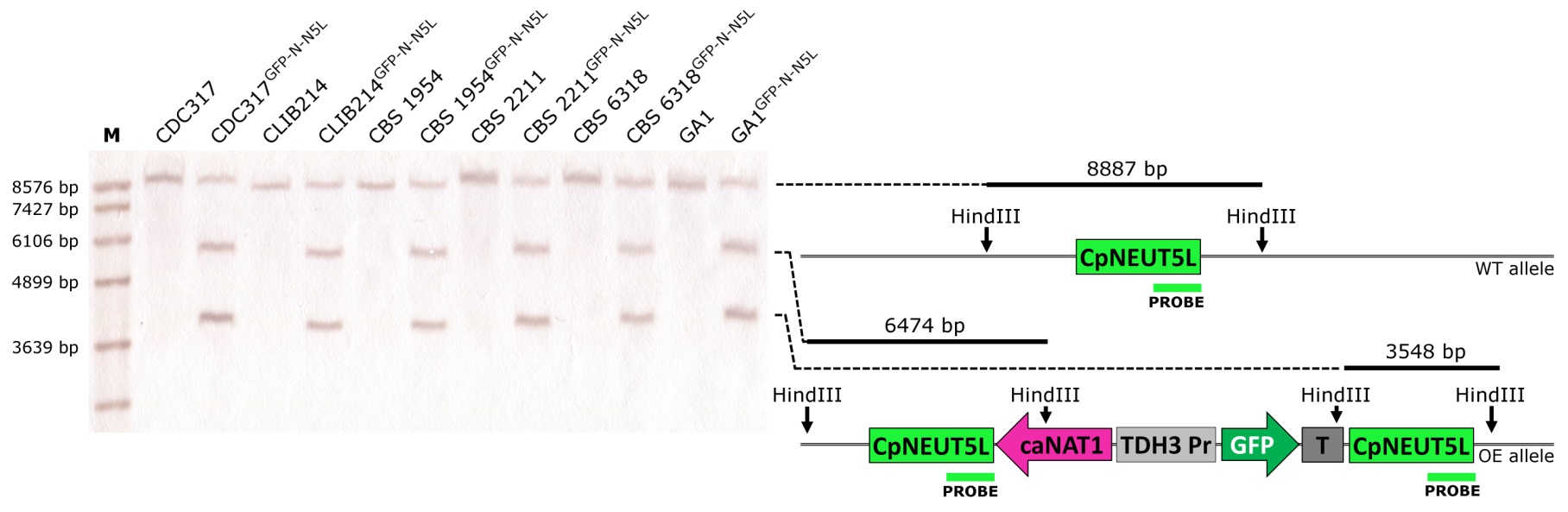
**

**Supplementary Figure S3.** Characterization of GFP-expressing strains of prototroph *C. parapsilosis* isolates by flow cytometer. Every row represents a given prototroph isolate **(A**, **D** and **G)** its GFP-labeled derivative generated by using either pECpOE-GFP-N-N5L **(B**, **E** and **H)** or pNRVL-S-GFP **(C**, **F** and **I)**.


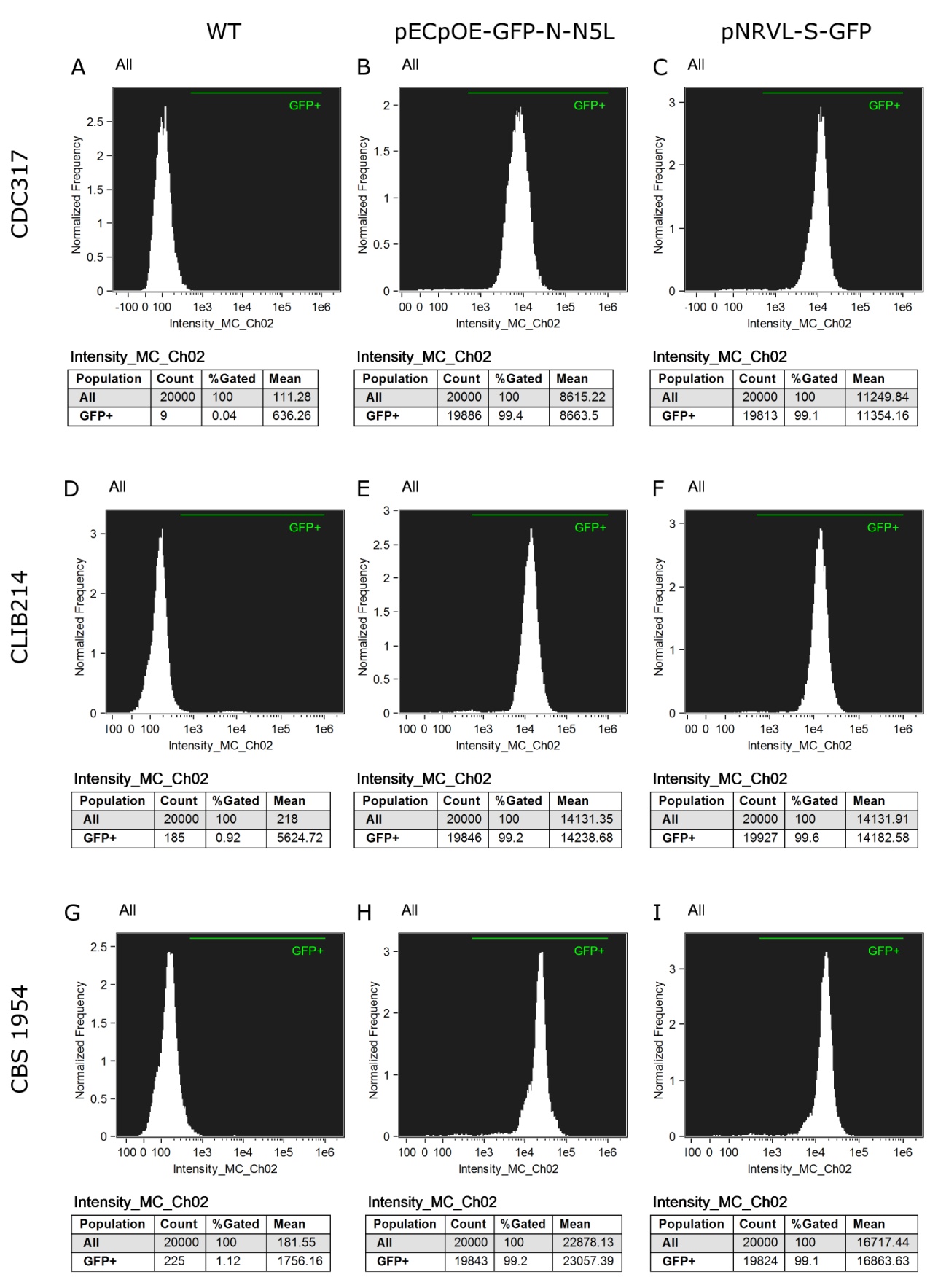


**Supplementary Figure S4.** Characterization of GFP-expressing strains of prototroph *C. parapsilosis* isolates by flow cytometer. Every row represents a given prototroph isolate **(A**, **D** and **G)** its GFP-labeled derivative generated by using either pECpOE-GFP-N-N5L **(B**, **E** and **H)** or pNRVL-S-GFP **(C**, **F** and **I)**.


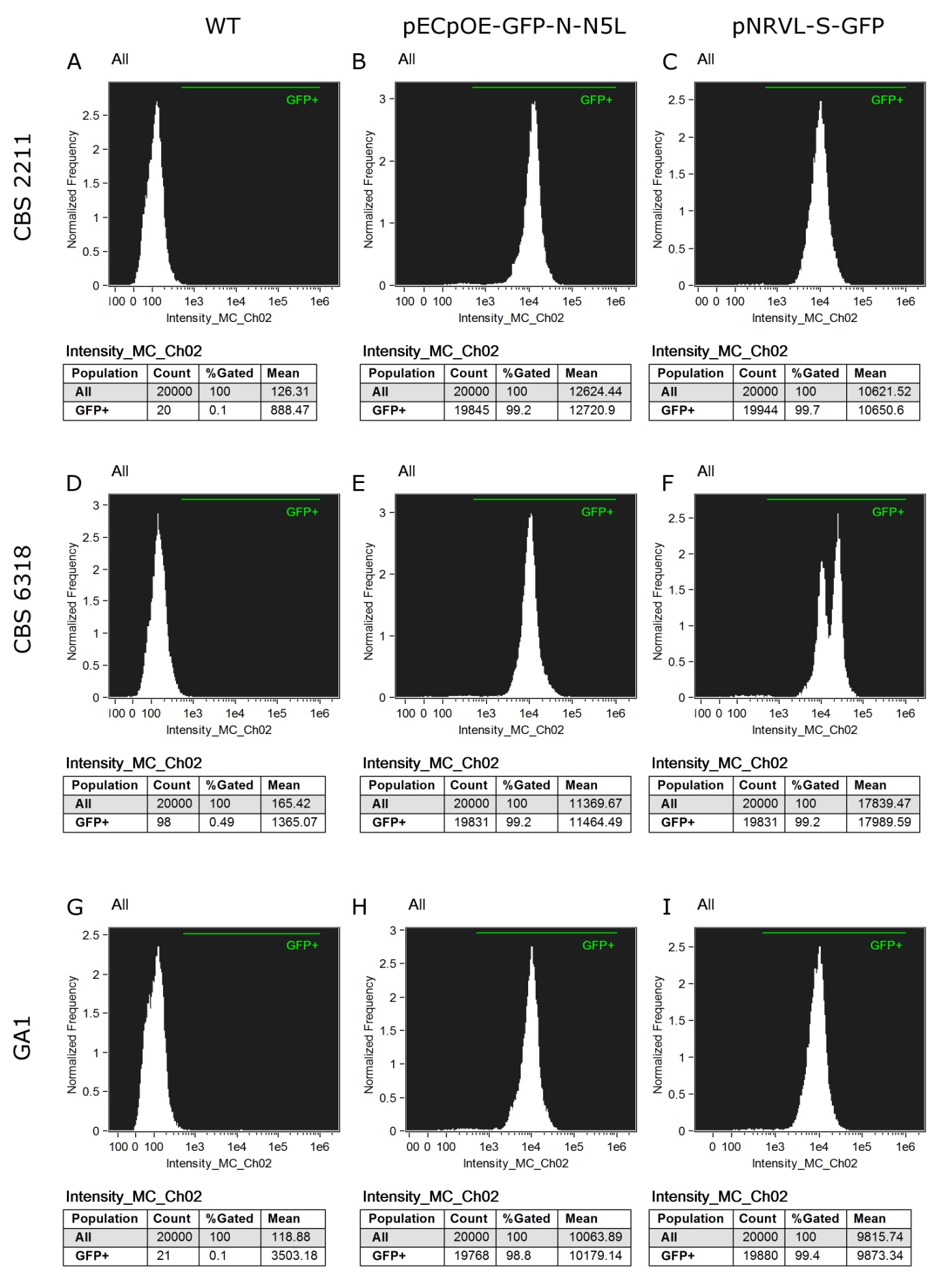


**Supplementary Figure S5**. Validation of the GFP-expressing transformants generated with pNRVL-S-GFP by PCR. **(A)** shows the conception of the validation with primers specific to the genomic DNA (solid arrows) or to the plasmid sequence (empty arrows). GFP-labeled derivatives of CDC317, CLIB214 **(B)**, CBS 1954, CBS 2211 **(C)** and CBS 6318, GA1 **(D)** were generated.


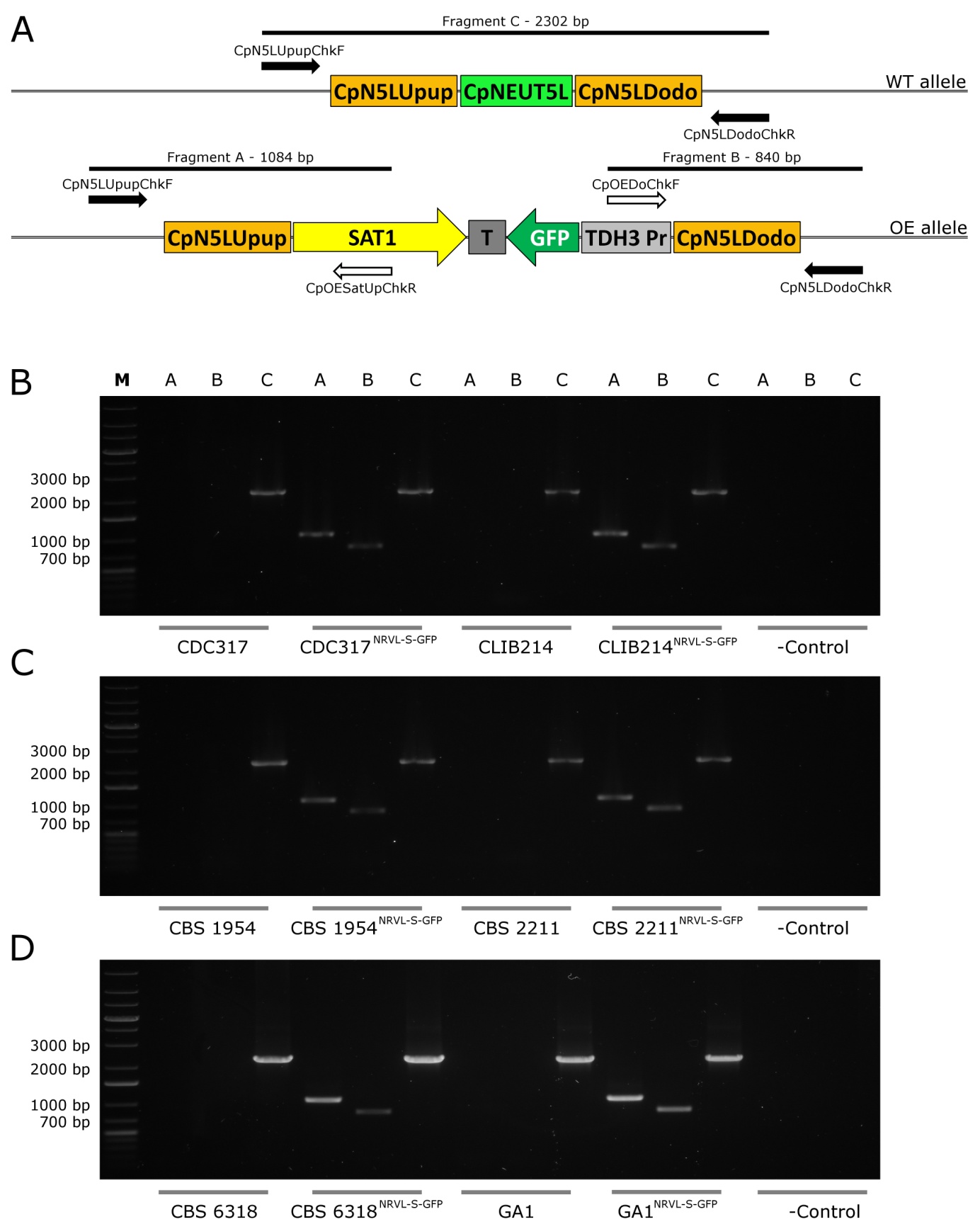


**Supplementary Figure S6.** Validation of the GFP-expressing transformants by Southern-blot. Six prototroph *C. parapsilosis* isolates were GFP-labeled by using pNRVL-S-GFP.

**
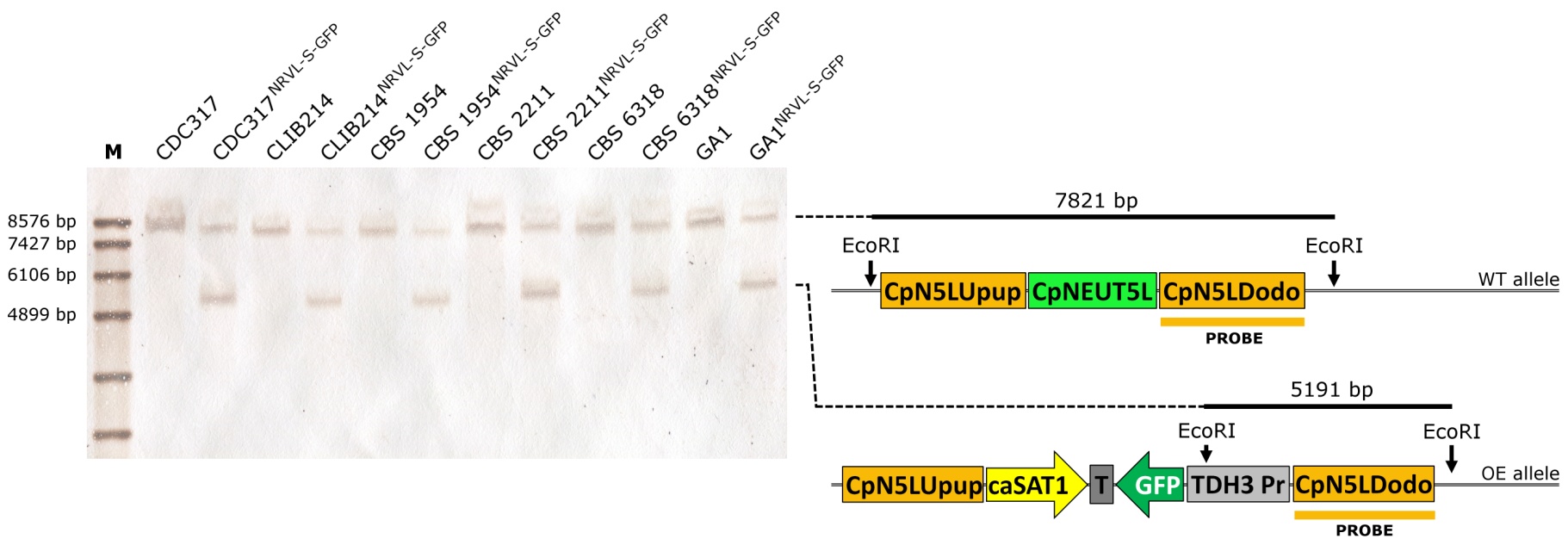
**

**Supplementary Figure S7.** Comparison of the GFP positive population of fungal cells generated by using auxotrophic or dominant selectable marker. Significant difference was found between the two groups independently of the vector (pNRVL or pCpOE) applied.


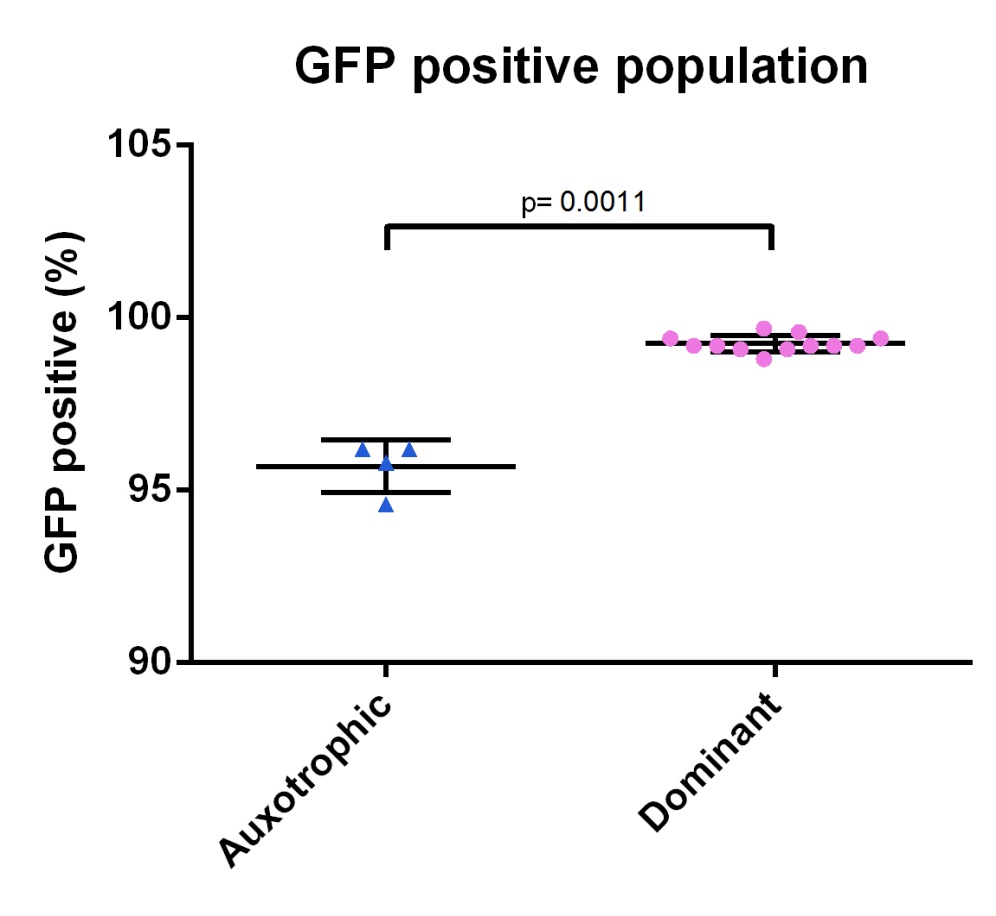


**Supplementary Figure S8.** Comparison of the fitness of the mutants on solid media. CPRI, CPL2 control - and CPL2^GFP-L-N5L^ /1 and /2 GFP-expressing strains were grown on solid media representing different stress conditions. Plates were incubated for two days at 30 C°.

**
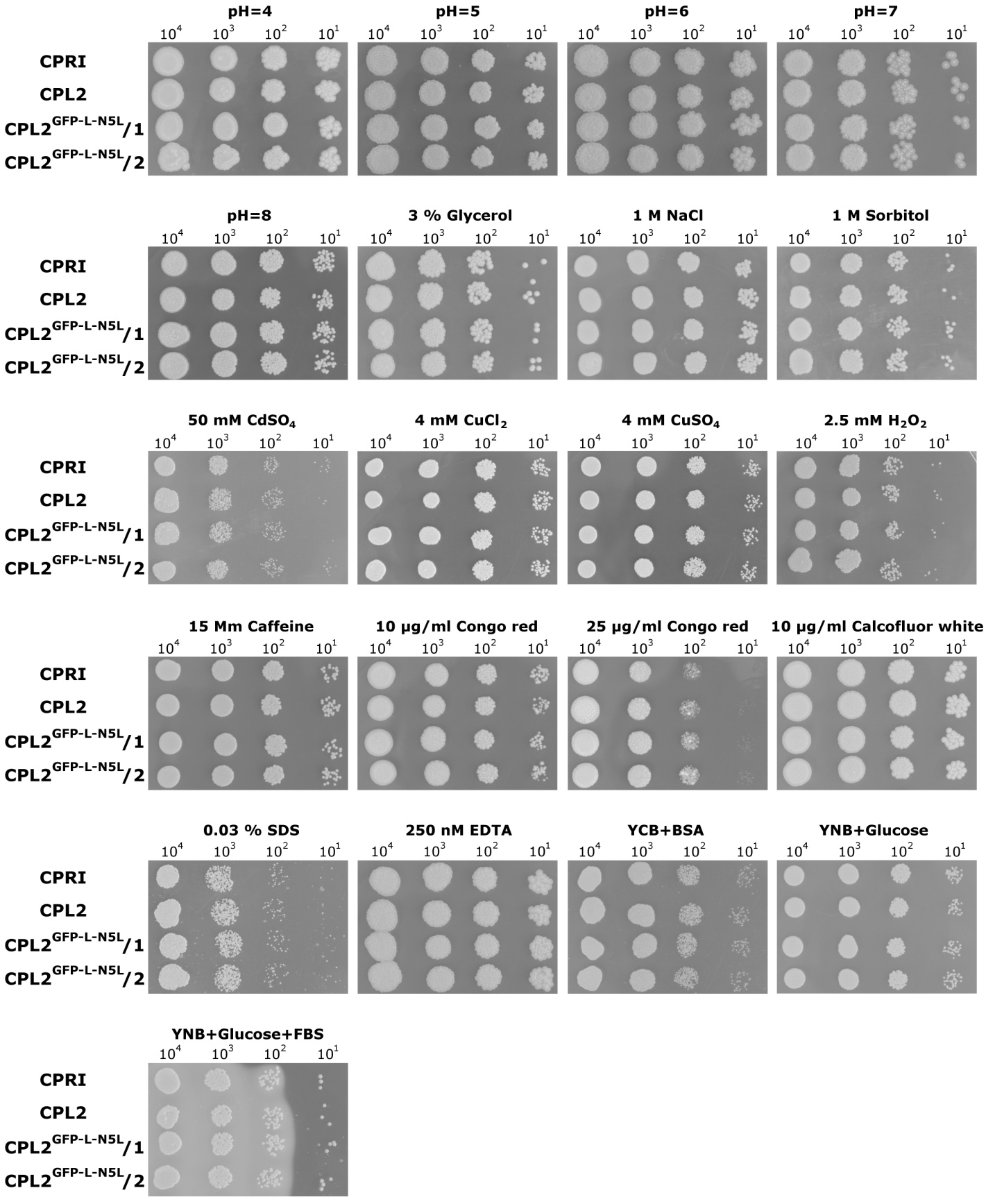
**

**Supplementary** **Figure S9.** Comparison of the fitness of the mutants on solid media. CPRI, CPL2 control - and CPL2^GFP-L-N5L^ /1 and /2 GFP-expressing strains were grown on solid media representing different stress conditions. Plates were incubated for two days at 37 C° except CdSO_4_ that was kept at 37 °C for four days.

**
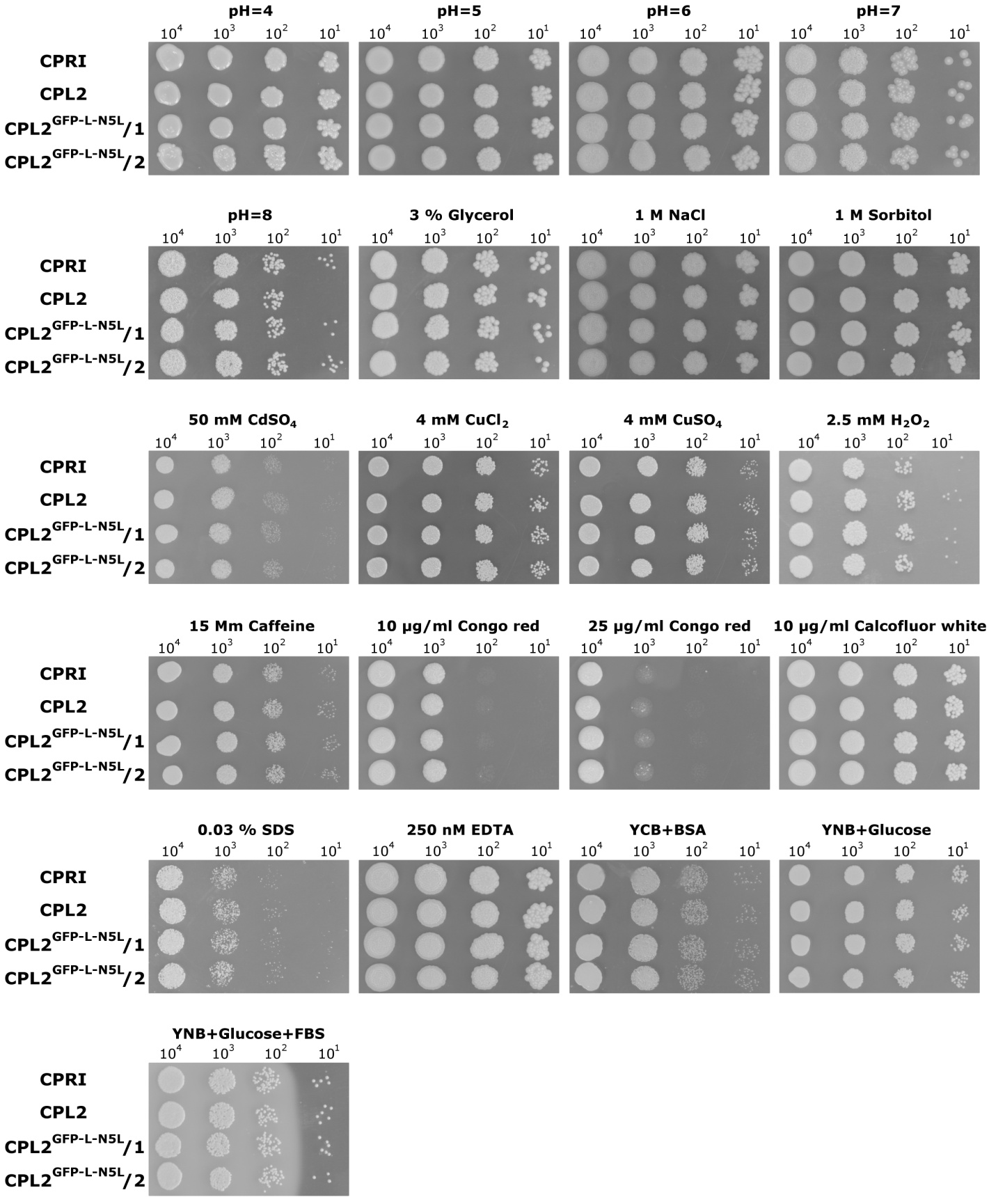
**

**Supplementary Figure S10.** Comparison of the fitness of the mutants on solid media. CPRI, CPL2 control - and CPL2^NRVL-L-GFP^ /1 and /2 GFP-expressing strains were grown on solid media representing different stress conditions. Plates were incubated for two days at 30 C°.


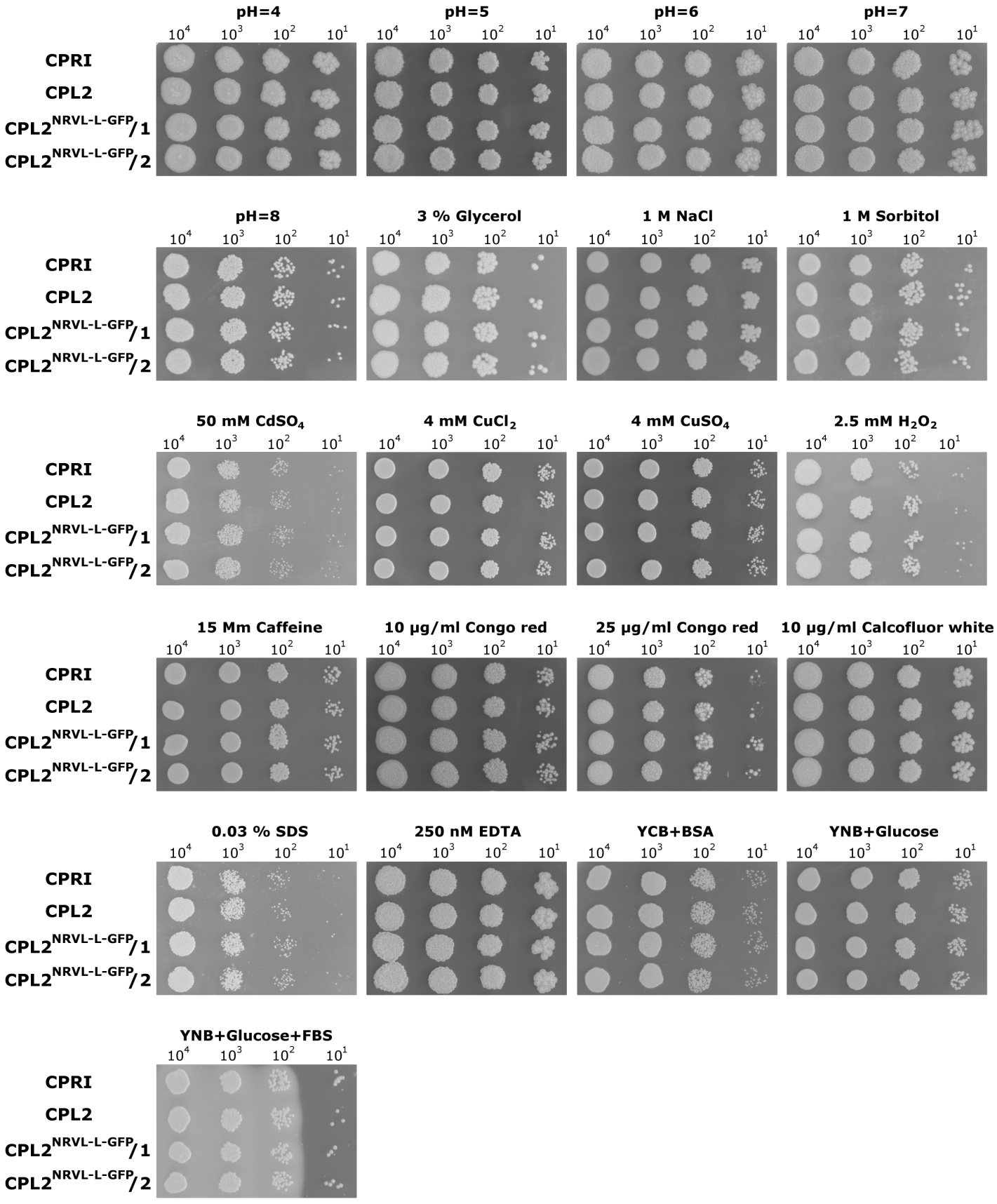


**Supplementary Figure S11.** Comparison of the fitness of the mutants on solid media. CPRI, CPL2 control - and CPL2^NRVL-L-GFP^ /1 and /2 GFP-expressing strains were grown on solid media representing different stress conditions. Plates were incubated for two days at 37 C°, except CdSO_4_ that was kept at 37 °C for four days.


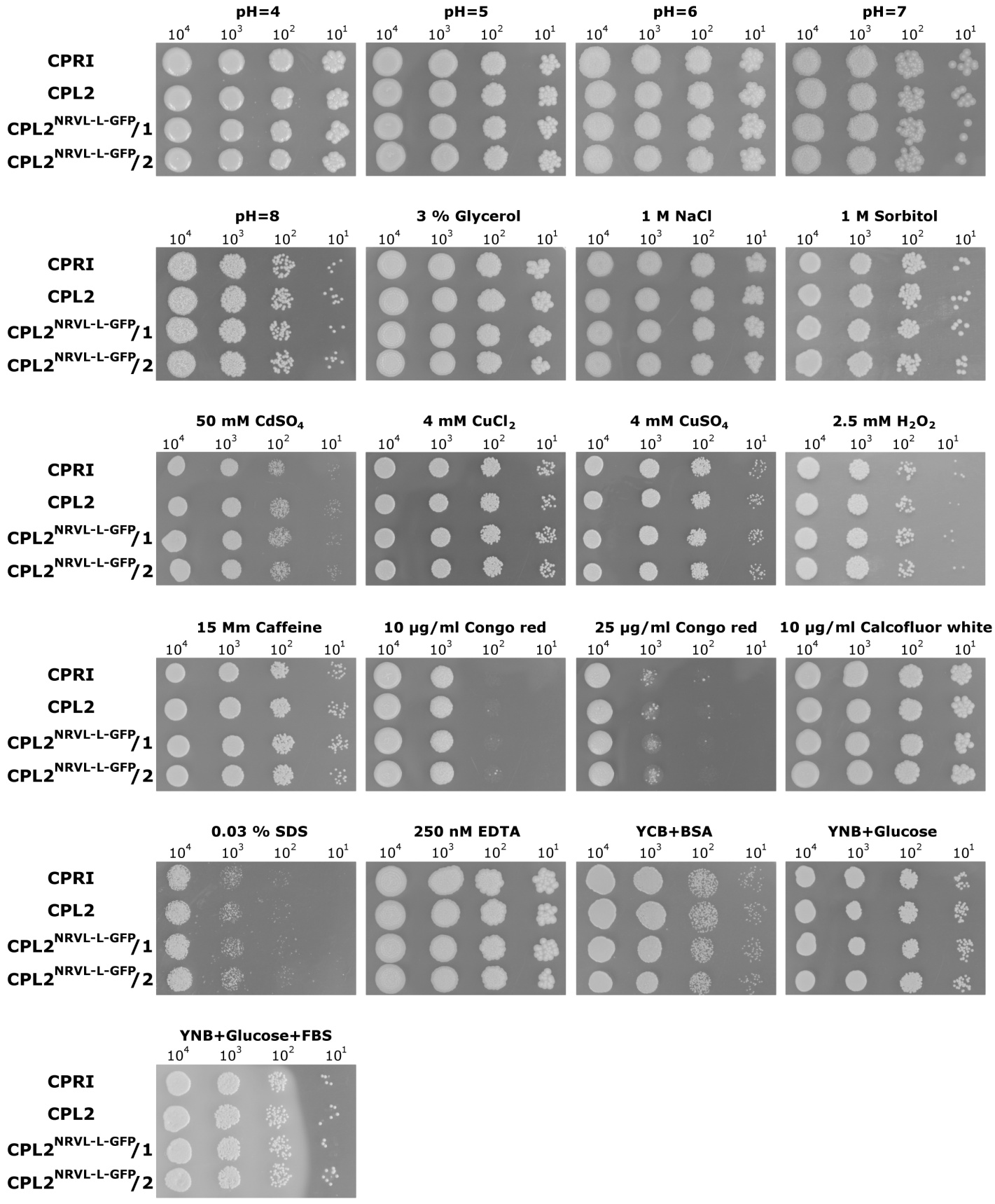


**Supplemetary Figure S12.** Comparison of the fitness of the mutants in liquid culture. CPRI, CPL2 control - and CPL2^GFP-L-N5L^ /1 and /2 GFP-expressing strains were grown at 30 °C in liquid media representing different stress conditions. OD_600_ was measured in every other hour.


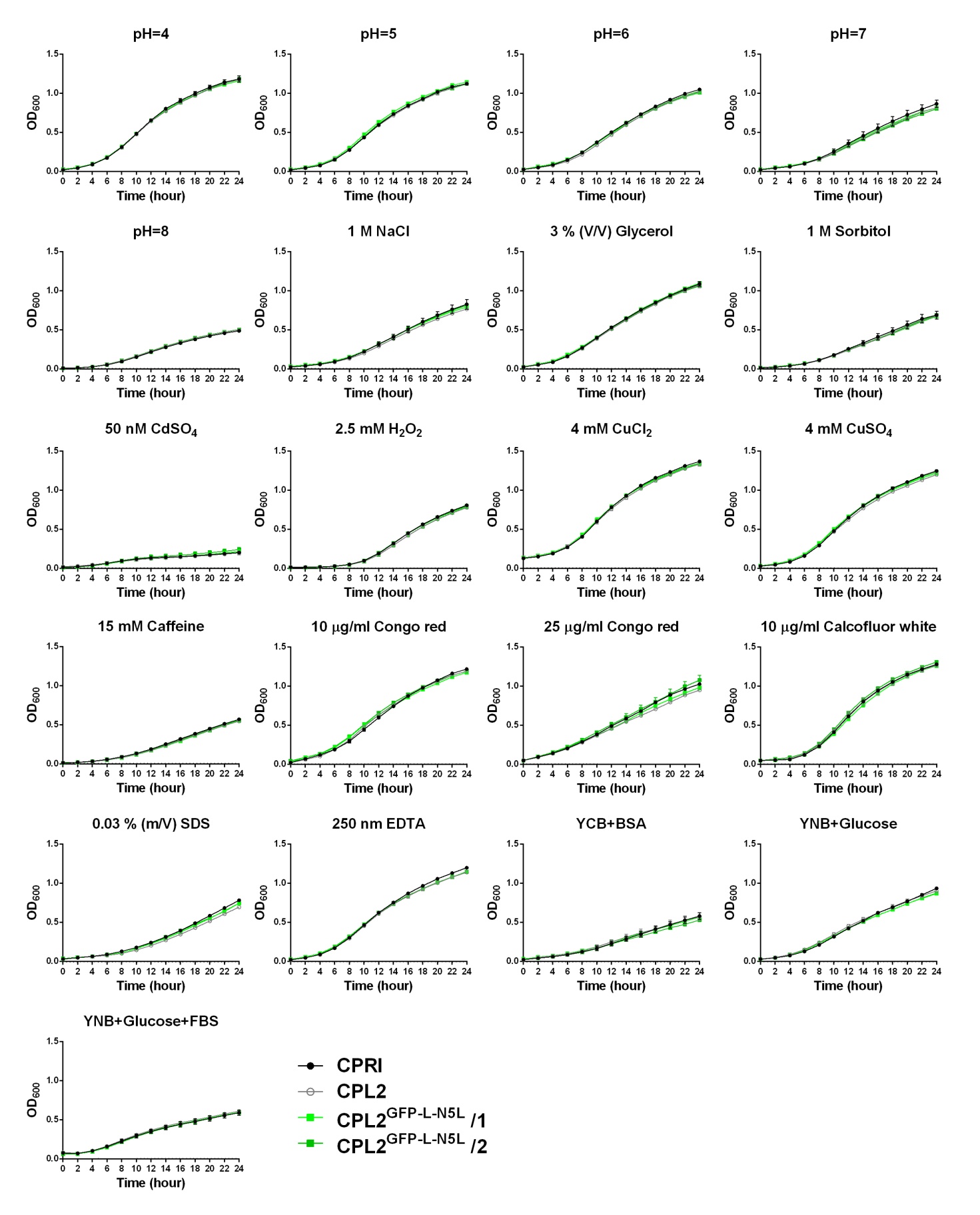


**Supplementary Figure S13.** Comparison of the fitness of the mutants in liquid culture. CPRI, CPL2 control - and CPL2^GFP-L-N5L^ /1 and /2 GFP-expressing strains were grown at 37 °C in liquid media representing different stress conditions. OD_600_ was measured in every other hour.


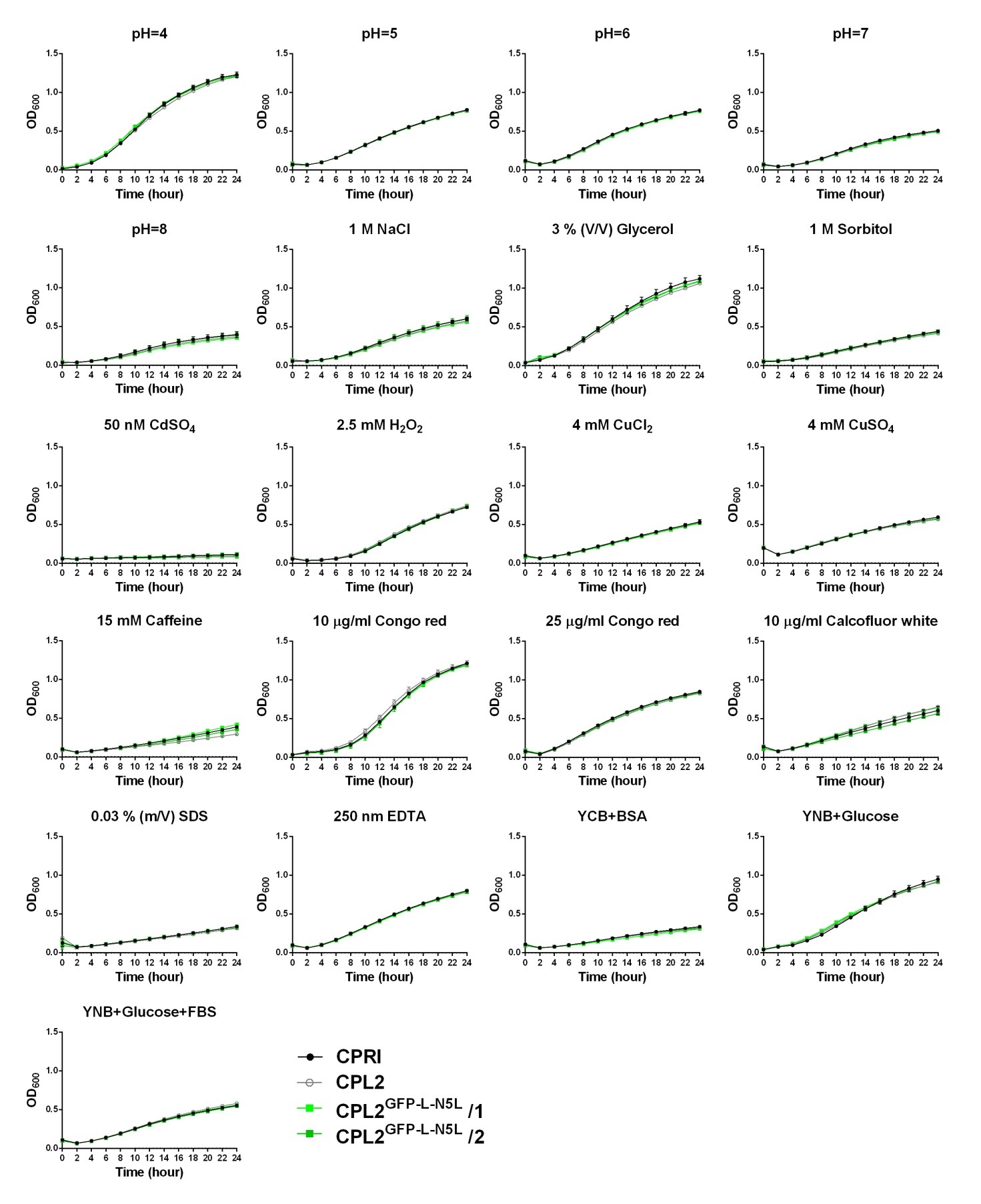


**Supplementary Figure S14.** Comparison of the fitness of the mutants in liquid culture. CPRI, CPL2 control - and CPL2^NRVL-L-GFP^ /1 and /2 GFP-expressing strains were grown at 30 °C in liquid media representing different stress conditions. OD_600_ was measured in every other hour.


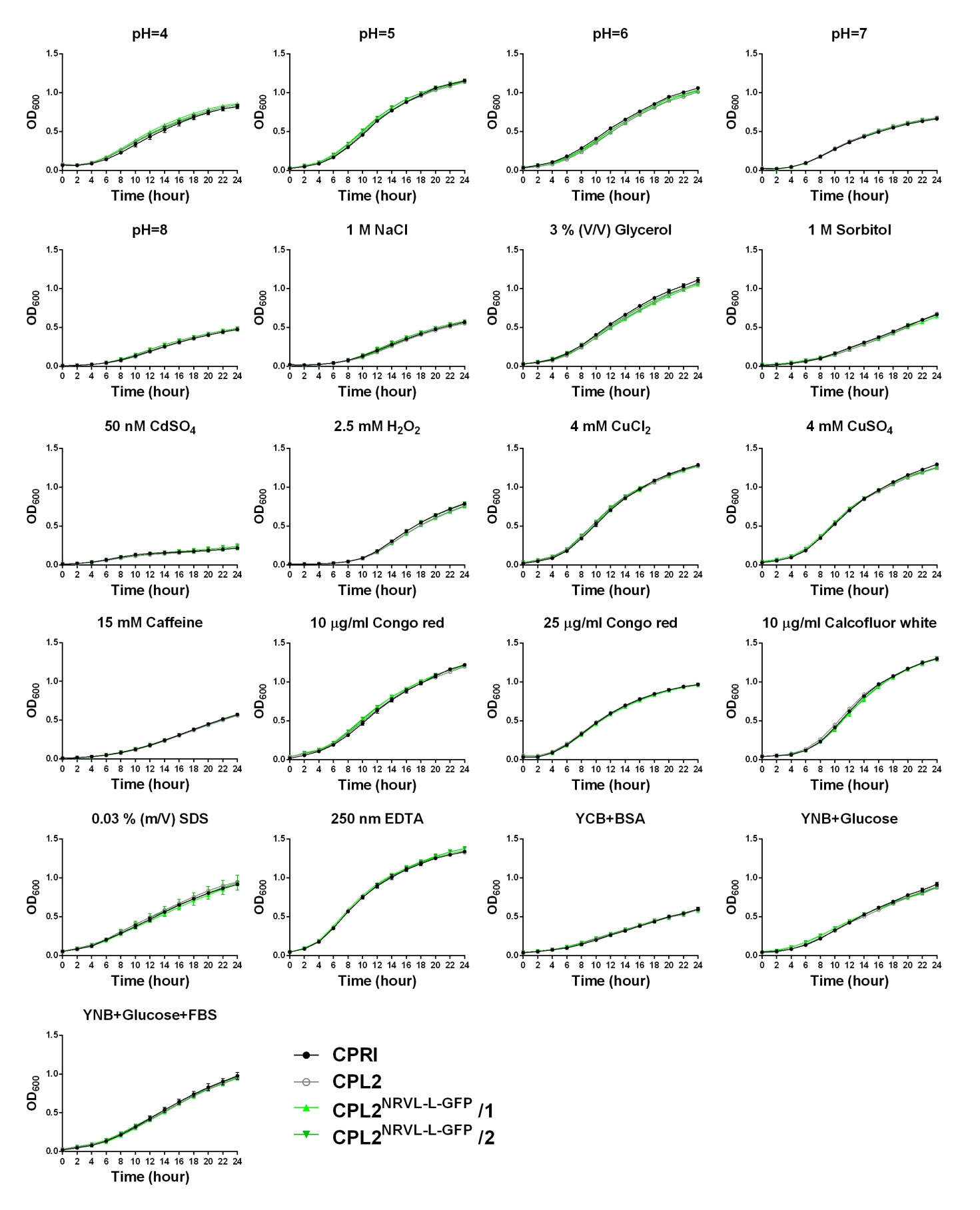


**Supplementary Figure S15.** Comparison of the fitness of the mutants in liquid culture. CPRI, CPL2 control - and CPL2^NRVL-L-GFP^ /1 and /2 GFP-expressing strains were grown at 37 °C in liquid media representing different stress conditions. OD_600_ was
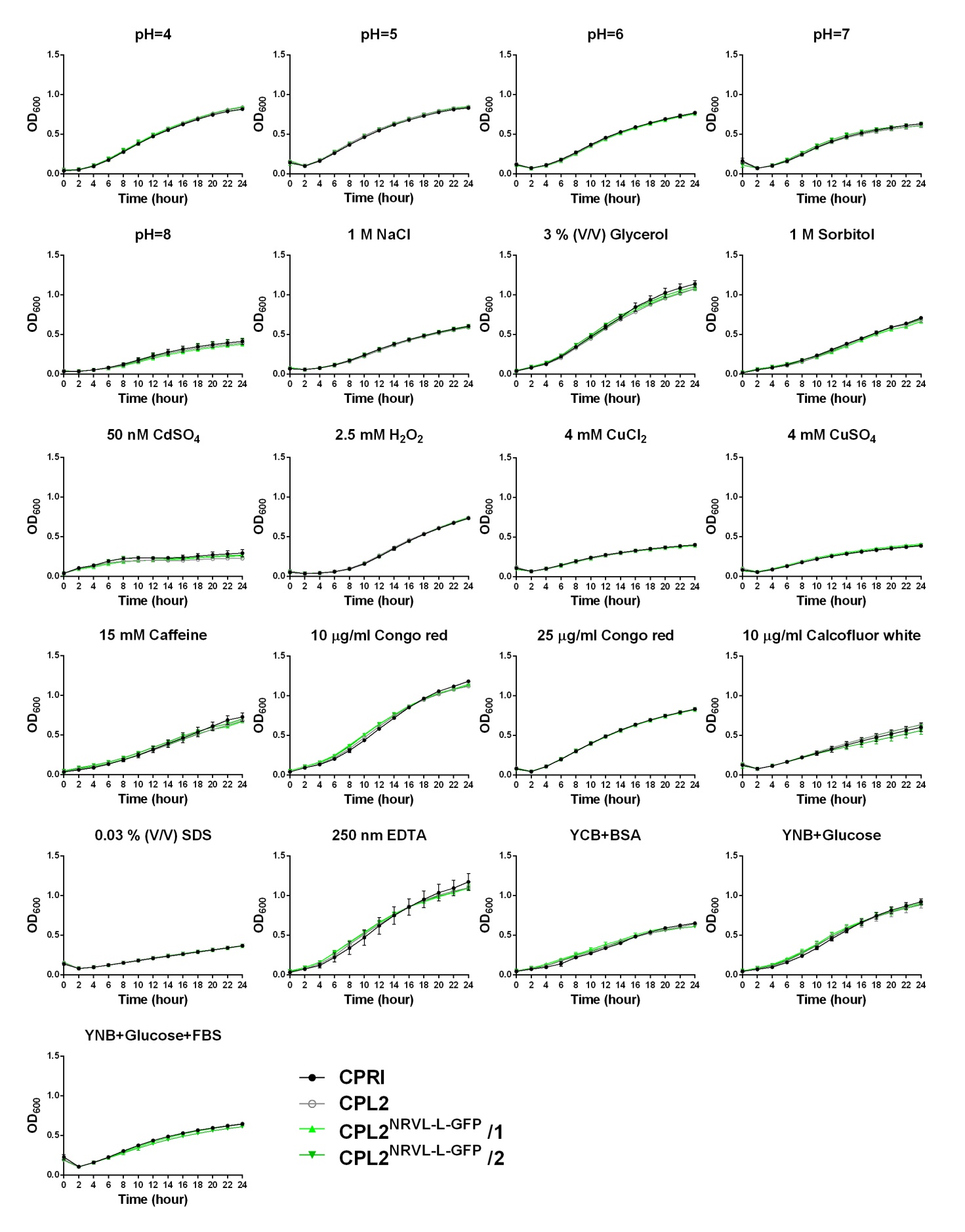
measured in every other hour.

**Supplementary Figure S16.** Validation of *ade2^-/-^* and ADE2^RI^ mutants by PCR. **(A)** shows the conception of the validation with primers specific to the genomic DNA (solid black arrows) or to the plasmid sequence (dark/light blue arrows). Note that due to the two selection markers there are two Fragment A and B, one for the HIS1 and one for the LEU2 cassette. **(B)** presents the validation of the ADE2^RI^ mutant. Solid arrows show primers specific to the genome, empty arrows indicate primers specific to the integrated sequence. CPRI was used as a control in the experiments, CPL2H1 is the parental strain of *ade2^-/-^* and ADE2^RI^ is its reintegrant derivative.


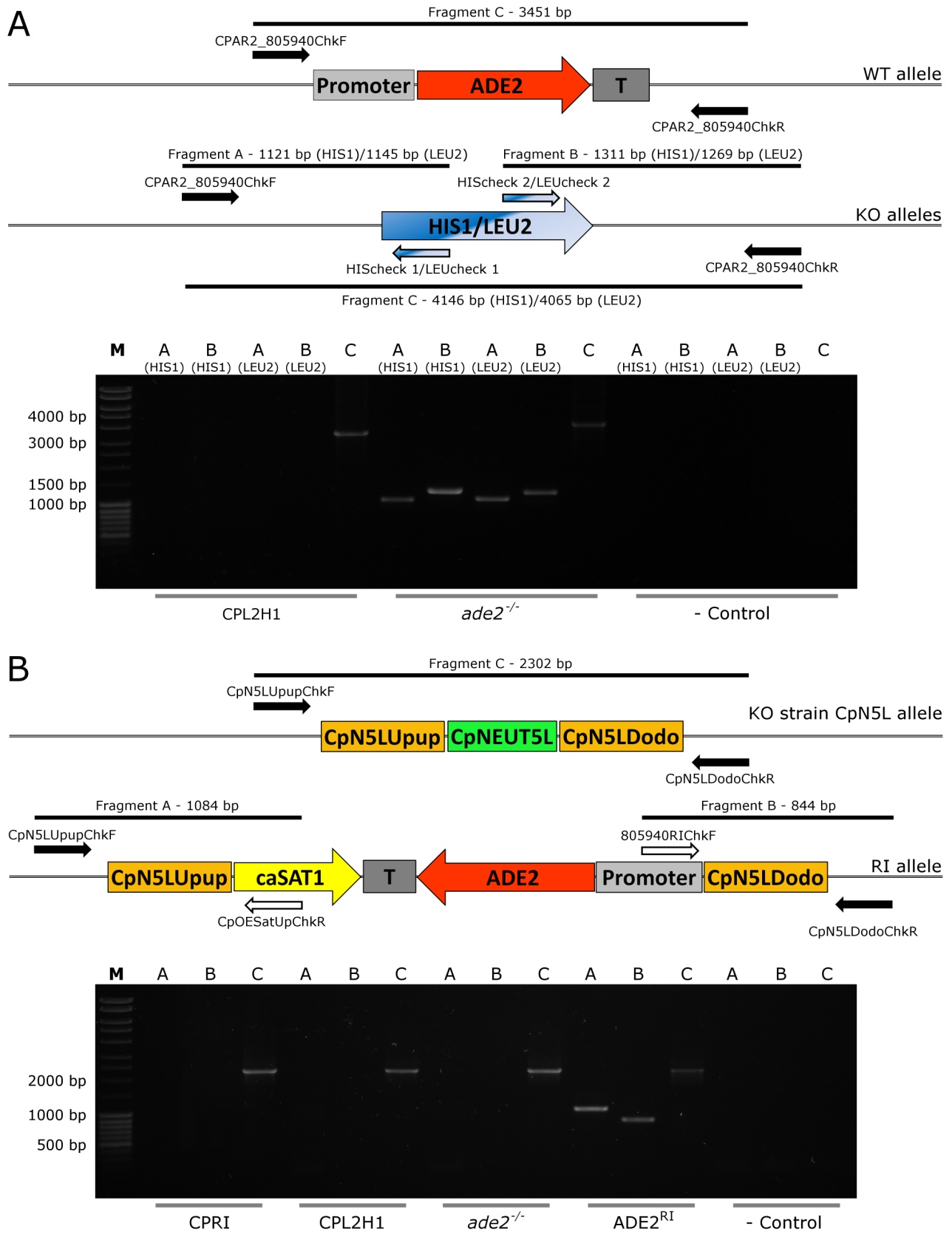


**Supplementary Figure S17.** Validation of *ade2^-/-^* and ADE2^RI^ transformants by Southern-blot. Scheme describes the genetic background of CPRI (control strain of the experiments), CPL2H1 (parental strain of *ade2^-/-^), ade2^-/-^* and ADE2^RI^ reintegrant strain. *HIS1* and *LEU2* carrying cassettes used for knocking out *ADE2* gene possess different digestion patterns. Note that scheme explaining the concept of the validation is not drawn to scale.


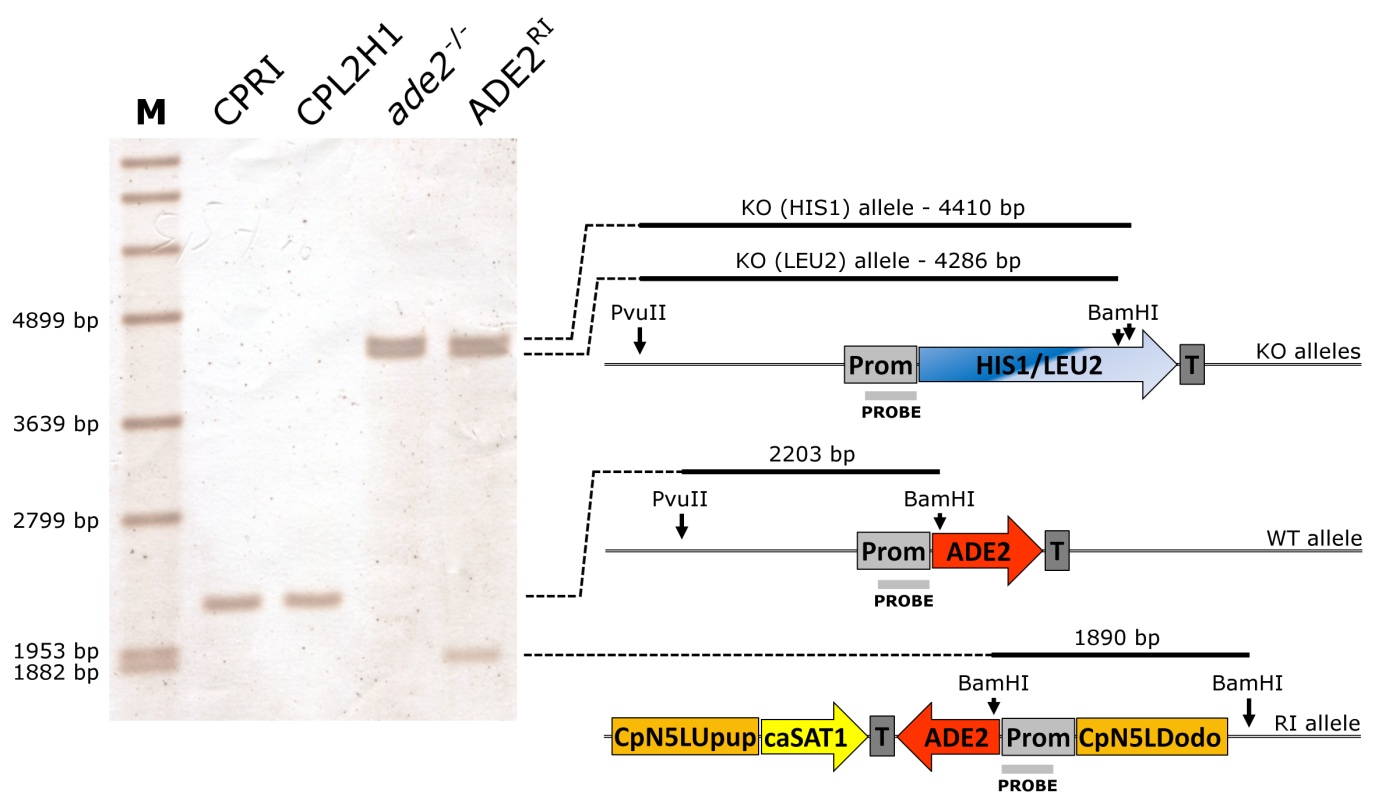

Supplement: Supplementary file 1 [file Data_Sheet_1.docx]
